# In-depth Proteomic mapping of mouse (*Mus musculus*) epididymal constructive basis for sperm maturation

**DOI:** 10.1186/s12953-015-0076-3

**Published:** 2015-07-30

**Authors:** Xin Liu, Fu-Jun Liu, Shao-Hua Jin, Xiao-Fang Shen, Yan-Wei Wang

**Affiliations:** Central Laboratory, Yantai Yu Huang Ding Hospital/Qingdao University, Yantai, 264000, Shandong People’s Republic of China; Clinical Laboratory, Yantai Yu Huang Ding Hospital/Qingdao University, Yantai, 264000, Shandong People’s Republic of China

**Keywords:** Sperm maturation, Structural proteins, Mouse epididymis, Bioinformatics, Proteomics

## Abstract

**Background:**

The mouse epididymis performs an essential role in sperm maturation, but global protein expression data in mouse epididymis are still lacking. Here, we reported the first in-depth gel-based profiling of mouse epididymis proteome and established a 2-DE map.

**Results:**

A total of 832 protein spots were detected in the reproducible gels, and 625 spots corresponding to 355 unique protein entries have been successfully identified by MALDI-TOF-MS. The confidence of proteome data was validated by Western blot. Functional annotations showed that these proteins were mainly related to general metabolism, antioxidant and structural molecule activity. Immunohistochemistry disclosed two structural proteins (myosin regulatory light polypeptide 9 and alpha-2 type I collagen) continuously expressed in the myoid cell since postpartum.

**Conclusion:**

This study provides a first-draft reference map of the mouse epididymis proteome, which will greatly expand the knowledge of the epididymal structural basis and contribute to the better understanding of those proteins in the process of mouse epididymal sperm maturation.

**Electronic supplementary material:**

The online version of this article (doi:10.1186/s12953-015-0076-3) contains supplementary material, which is available to authorized users.

## Introduction

The epididymis is a tightly elongated tube, which connects the testis with the vas deferens. Spermatozoa produced by the testis are not yet motile or fertile. During the epididymal transit, those immature spermatozoa interact with the epididymal molecules, especially with the epididymal proteins [1]. Then the sperm surfaces undergo complex modifications, such as the loss or increase of the protein composition, and the glycosylation or phosphorylation modifications [2, 3]. Finally, these immature spermatozoa acquire the maturational capacity to survive in the female tract, move to the oviduct, penetrate the egg, and fuse with the oocyte [4]. Therefore, the epididymis is the vital organ for sperm maturation. Several important epididymal proteins have been described, such as clusterin [5], Bin1b [6], CD52 [7], ADAM7 [8], but more proteins were still expected to be identified for understanding the epididymal roles in spermatozoa maturation.

Epididymal proteomes of several species have been performed such as boar [9], stallion [10], ram [11], bovine [12] and human [13]. The results identified few common epididymal proteins across different species. Therefore, the species-specific epididymal proteins exist and cannot be ignored. In this paper, we presented an in-depth two-dimensional epididymal proteome map of the mouse *Mus musculus*. The result would provide important information for understanding the mouse epididymal functions.

## Results and discussion

**Fig. 1:**
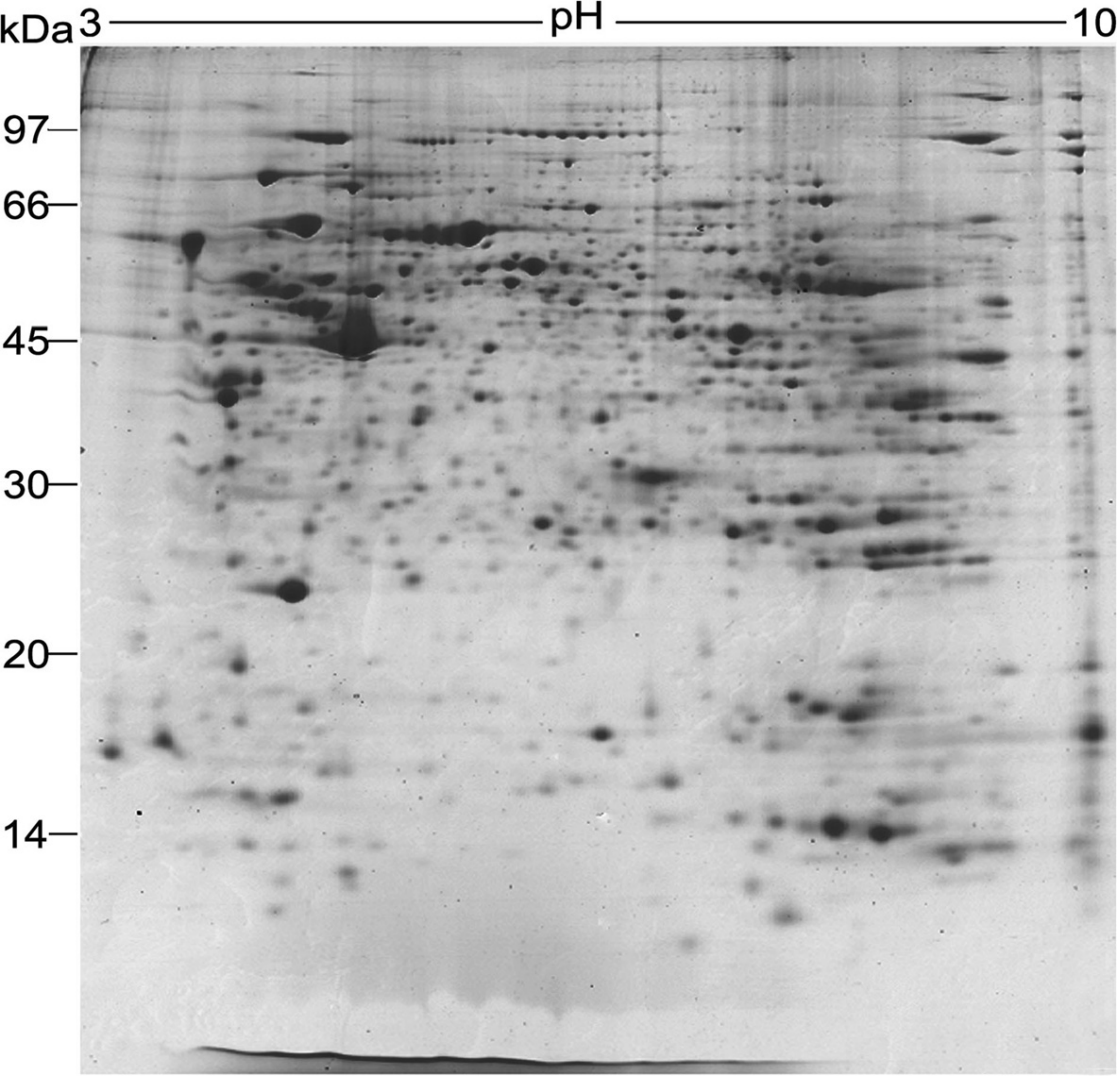
A representative 2D gel of the mouse epididymal proteins

Eight hundred and thirty two spots were consistently present on the two-dimensional gel (Additional file 1: Figure S1). Fig. 1 and Additional file 2: Figure S2 showed the representative two-dimensional gel0 and an annotated gel respectively. All the 832 spots were excised, digested, and analyzed by mass spectrometry. Six hundred and twenty-five spots (75.1 %) out of 832 spots were confidentially identified, and among them, 119 unique proteins (33.5 %) were identified from more than one spot in the gel (Additional file 3: Table S1 and Additional file 4: Figure S3), 625 spots corresponded to 355 unique proteins. The molecular masses were from 8.1 to 309.2 kDa, and their isoelectric points varied between 3.86 and 11.46.

The data confidence of mass spectrometry was validated by the analysis of western blot, and the results were in accordance with the proteomic data (Additional file 5: Figure S4). All the identified proteins were categorized by the Gene Ontology (GO) annotations, which included three aspects: molecular function, cellular component, and biological process.

**Fig. 2:**
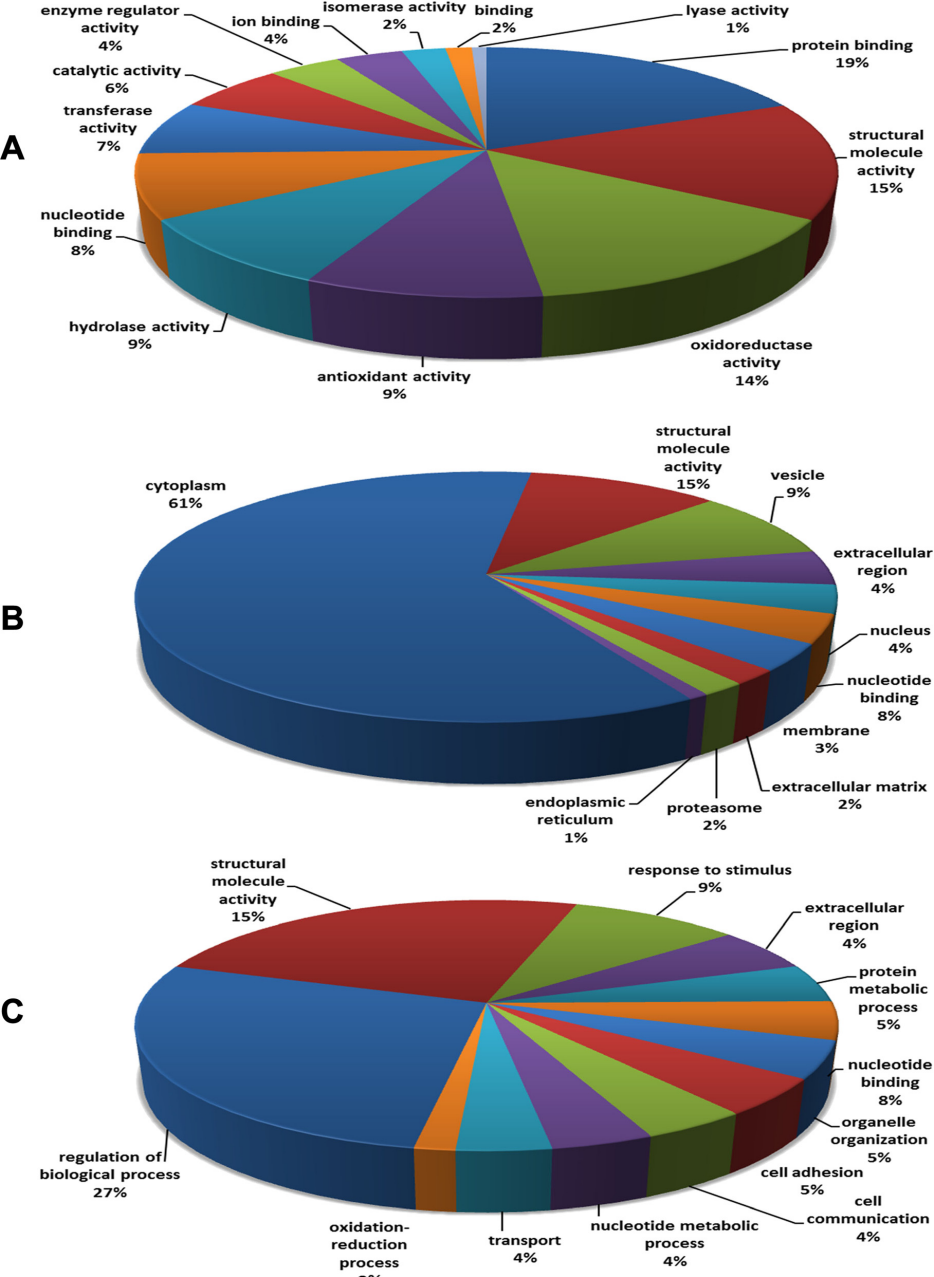
Classification of the mouse epididymal proteins based on gene ontology annotations for **a** Molecular Functions, **b** Cellular Component, and **c** Cellular Process

**Fig. 3:**
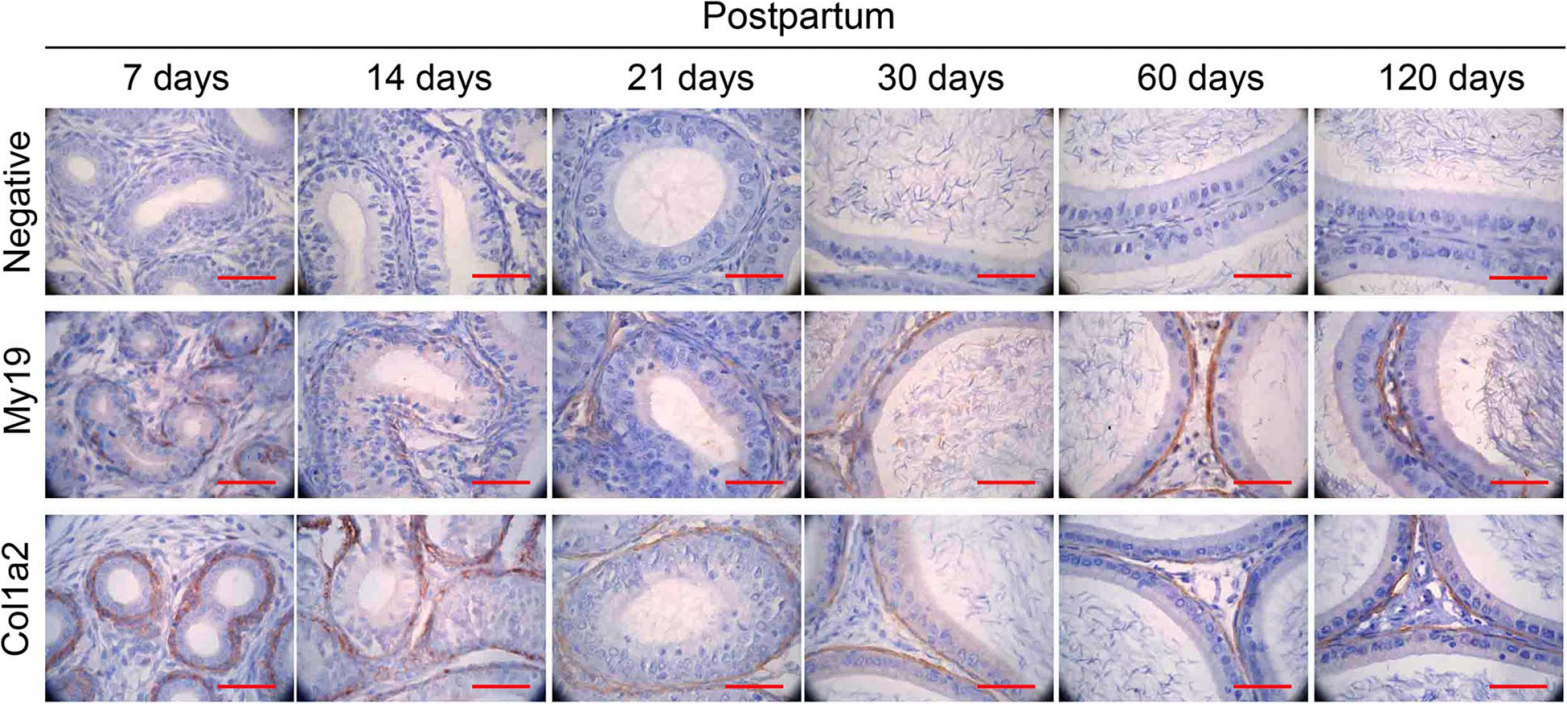
Immunohistochemistry analysis of myosin regulatory light polypeptide 9 (My19) and alpha-2 type I collagen (Col1a2) at 6 time points (days 7, 14, 21, 30, 60 and 120 postpartum) in the corpus of mouse epididymis. Each bar was 40μm

Annotations for molecular function, 313 (90.7 %) mouse epididymal proteins were assigned to 89 different functions, and then they were clustered into 13 GO slim categories (Fig. 2a). The largest part was protein binding (19 %), followed by structural molecule activity (15 %), oxidoreductase activity (14 %), antioxidant activity (9 %), hydrolase activity (9 %), nucleotide binding (8 %), transferase activity (7 %), catalytic activity (6 %), enzyme regulator activity (4 %), and ion binding (4 %). The GO slims isomerase activity (2 %), and lyase activity (0.8 %) were the smaller part. Those proteins (15 %) with structural molecule activity might be involved in the formation of the tight junction of blood-epididymis barriers, which regulated the exchange of molecules between the epididymal lumen and the blood circulation, and created a specific epididymal microenvironment for sperm maturation [14, 15]. Additionally, almost 10 % proteins were related to antioxidant activity, which removed excessive reactive oxygen species to provide a non-oxidative environment and protect epididymal cell DNA from oxidative damage [16, 17]. Molecular function showed the structural-related proteins were a main part in the whole identified proteins. Immunohistochemistry analysis (Fig. 3) also indicated the structural proteins (myosin regulatory light polypeptide 9 and alpha-2 type I collagen) indeed existed at different development stages since postpartum, which were around the epididymal tubule [18, 19]. Those structure proteins might provide the structural basis for the maintenance of epididymal sperm maturation environment, the epididymal contraction and the transport of epididymal sperm [18].

Annotations for the cellular compartments were founded for 266 (77.1 %) mouse epididymal proteins. Those proteins were allocated to 75 cellular compartments and were classified into 10 GO slim categories (Fig. 2b). Most terms were assigned to cytoplasm (61 %). Cytoskeleton, vesicle, extracellular region, nucleus, membrane, extracellular matrix, and proteasome for 9 %, 4 %, 4 %, 3 %, 2 %, and 28 % GO terms, respectively. The smallest fraction was localized to endoplasmic reticulum (1 %). The most fraction of cytoplasm (61 %) demonstrated the methodology bias of two-dimensional gel, which was inclined to the identification of cytoplasmic and soluble proteins [20].

For biological process, 285 proteins (82.6 %) were annotated by 228 cellular processes, and then were divided into 12 GO slim categories (Fig. 2c). The major biological process term was regulation of biological process (27 %). The remaining terms were designed to protein metabolic process (5 %), cell adhesion (5 %), organelle organization (5 %), and transport (4 %). A small fraction was belonged to the oxidation-reduction process (2 %). More proteins related to regulation of biological process might meet the requirements of active absorption and secretion functions of epididymal epithelium [2, 21].

## Conclusions

This work provided the first two-dimensional map of the mouse epididymal proteome, greatly expanded the knowledge of the structural basis of the mouse epididymal sperm maturation, and facilitate biological interpretation of epididymal function in a network context. Our interest focused on proteins of the mouse epididymis because they are necessary for sperm maturation and may become potential post-testicular contraceptive targets.

## Materials and methods

### Animal and protein extraction

Ten adult Kunming mice (*Mus musculus*) were used, and all the experiments were approved by the Institutional Animal Care and Use Committee of Yantai Yu-Huang-Ding Hospital. Under anesthesia with diethyl ether, the mice were euthanized. The mice epididymides were immediately separated without blood vessels and connective tissues. Then, the mice epididymal tubules were minced with gentle pressure in the phosphate saline buffer. After agitation at 4 °C for 15 min, the tubules were collected by centrifugation at 130 × g for 10 min at 4 °C. The supernatant was removed and the tubule fragments were washed at 36 × g for 10 min at 4 °C for several times with phosphate saline buffer until no spermatozoa were found in the microscopic examination of the pellet medium. Sperm-free pellets were powdered in liquid nitrogen and dissolved in the protein lysis solution containing 7 M urea, 2 M thiourea, 4 % CHAPS, 65mM DTT at 4 °C for 2 h. After centrifugation at 40,000 × g for 1 h at 4 °C, the supernatant were transferred into a new fresh tube. Four volumes of ice-cold acetone were added into the tube and stored at −20 °C for 1 h. After centrifugation at 20,000 × g for 1 h at 4 °C, precipitates were washed with ice-cold acetone, air-dried, dissolved in the protein lysis buffer and stored at −80 °C until use. Bradford assay was used to determine the protein concentration [22].

### 2-DE and protein identification

Eighteen cm nonlinear pH 3–10 IPG strips were used for one-dimensional IEF. After reduction and iodoacetamide, the strips were run on 12.5 % (w/v) SDS-PAGE. The gels were stained with CBB R-350 (Amersham Biosciences, Buckinghamshire, England), and were scanned by the Z320 scanner (Founder, Beijing, China). The gel maps were analyzed with the software of Imagemaster 6.0 (GE Healthcare). The electrophoresis experiments were performed in triplicate. The gel spots were excised, destained with 25mM NH_4_HCO_3_/50 % (v/v) ACN, and digested by trypsin in 25mM NH_4_HCO_3_ at 37 °C for 12 h. The resultant peptides were analyzed by a Voyager DE-STR biospectrometry work station (Applied Biosystems/MDS SCIEX, Foster City, CA). The spectra data of mass spectrometry was searched against the NCBInr database (Swissprot Release 55.0; 356194 sequences; 127836513 residues) for *Mus musculus* (house mouse) with Mascot (http://www.matrixscience.com/, MatrixScience Ltd., UK). For peptide mass fingerprinting search, only if the protein score was more than 60 and the matched peptides was greater than or equal to 4, the protein was confirmed as a successful identification. If one gel spot corresponded to more than one protein, only the protein with the highest score was selected.

### Bioninformatics

The bioinformatics analysis was carried out as our previous report [13]. The identified proteins were retrieved against the PIR database (http://pir.georgetown.edu/pirwww/, accessed 12 August 2013). Gene ontological annotations (GO; http://www.geneontology.org/, accessed 12 August 2013) including molecular function, biological process and cellular component were clustered. Those without annotation were described as “unclassified”.

### Western blot

To further validate the identification confidence of proteins by mass spectrometry, nine proteins (clusterin; glyceraldehyde-3-phosphate dehydrogenase; peroxiredoxin-1; superoxide dismutase [Mn]; myosin regulatory light polypeptide 9; glutathione S-transferase P1; peroxiredoxin-6; alpha-2 type I collagen; superoxide dismutase [Cu-Zn]) were randomly selected and detected by western blot. For Western blot, 50 μg of protein was separated by 12.5 % (w/v) SDS-PAGE, and then was transferred to PVDF membranes. Subsequently, the membranes were blocked with 2 % (w/v) skimmed milk for 1 h and were incubated with the primary antibody at RT for 1 h. After washes by TBST for three times, membranes were incubated with HRP-conjugated anti-IgG for 1 h. A diaminobenzidene kit (Zhong Shan Biotechnology, Beijing, China) was used to visualize the immunoreactive complexes.

### Immunohistochemistry

The mouse epididymides were fixed in Bouin’s solution for 10 h, and embedded in paraffin. For antigen retrieval, the 4μm-thickness sections were processed in a microwave oven for 15 min, and 3 % (v/v) H_2_O_2_ was used to incubate the sections for 10 min to eliminate the endogenous peroxidases. After the antigen blocking with 3 % bovine serum albumin for 30min, primary antibody was added overnight at 4 °C. After TBS washed the sections for several times, horseradish peroxidase-conjugated anti-rabbit IgG (ZhongShan Biotechnology) was added for 1 h at 37 °C. The DAB kit (ZhongShan Biotechnology) was used to reveal the binding sites. Then the sections were counterstained by hematoxylin, and mounted with bright-field microscopy (DM LB2, Leica, Nussloch, Germany). For a negative control, pre-immune rabbit IgG was instead of primary antibody.

## Additional files

Additional file 1: Figure S1. The replicates of 2DE gels. (JPEG 3421 kb) Additional file 2: Figure S2. An annotated 2D gel of the mouse epididymal proteins. (JPEG 372 kb) Additional file 3: Table S1. 625 identified proteins of the mouse epididymis. (DOCX 139 kb) Additional file 4: Figure S3. The number of isoform or splice variants of the 355 mouse epididymal proteins. Most proteins were associated with only single spots on 2D gels. Fewer proteins had up to and over 5 spots. (JPEG 28 kb) Additional file 5: Figure S4. Western blotting of proteins found in mouse epididymis. Lane 1, clusterin; Lane 2, glyceraldehyde-3-phosphate dehydrogenase; Lane 3, peroxiredoxin-1; Lane 4, superoxide dismutase [Mn]; Lane 5, myosin regulatory light polypeptide 9; Lane 6, glutathione S-transferase P; Lane 7, peroxiredoxin-6; Lane 8, alpha-2 type I collagen; Lane 9, superoxide dismutase [Cu-Zn]; Lane 10, negative control. (JPEG 59 kb)

## Abbreviations

2-DE: Two-dimensional gel electrophoresis
ACN: Acetonitrile
CHAPS: 3-[(3-Cholamidopropyl) dimethylammonio]-1-propanesulfonate
DTT: Dithiothreitol
CBB: Coomassie brilliant blue
IEF: Isoelectric focusing
IPG: Immobilized pH gradient
MS: Mass spectrometry
RT: Room temperature
PVDF: Polyvinylidene fluoride
TBST: Tris-buffered saline Tween-20
