# Supplementary figures and images for: In-depth Proteomic mapping of mouse (Mus musculus) epididymal constructive basis for sperm maturation
Source: Proteome Sci. 2015 Jul 30;13:20. doi: 10.1186/s12953-015-0076-3 (PMC4518611; doi:10.1186/s12953-015-0076-3)

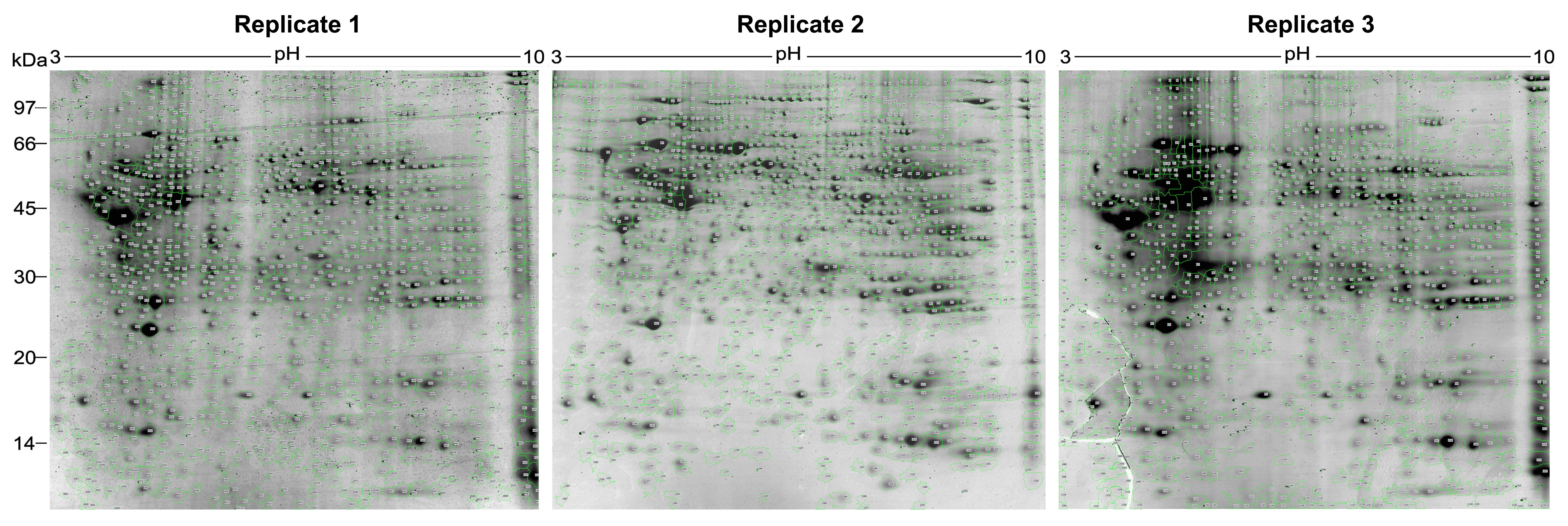

Supplement: Additional file 1: Figure S1. — The replicates of 2DE gels. (JPEG 3421 kb) [file 12953_2015_76_MOESM1_ESM.jpeg]

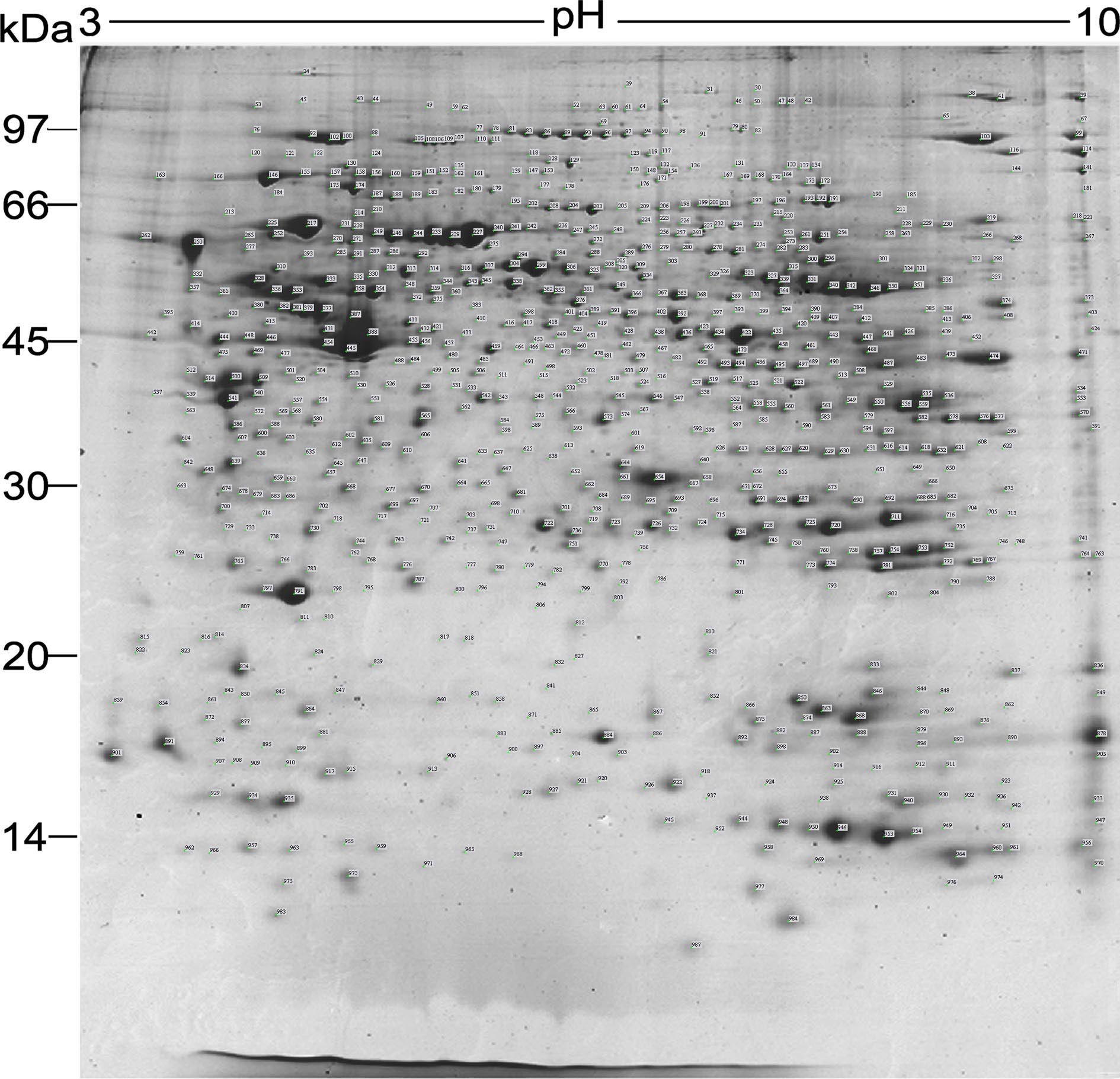

Supplement: Additional file 2: Figure S2. — An annotated 2D gel of the mouse epididymal proteins. (JPEG 372 kb) [file 12953_2015_76_MOESM2_ESM.jpeg]

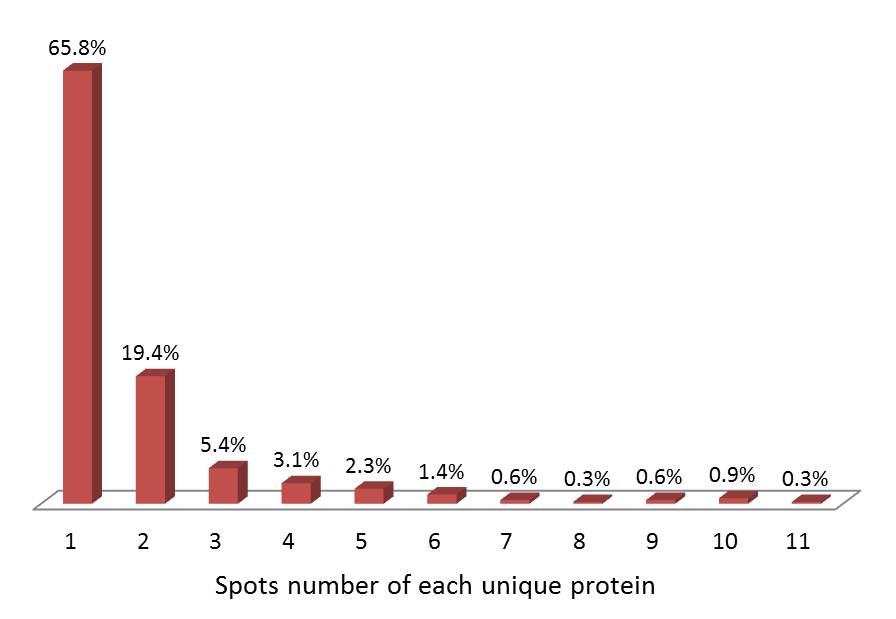

Supplement: Additional file 4: Figure S3. — The number of isoform or splice variants of the 355 mouse epididymal proteins. Most proteins were associated with only single spots on 2D gels. Fewer proteins had up to and over 5 spots. (JPEG 28 kb) [file 12953_2015_76_MOESM4_ESM.jpeg]

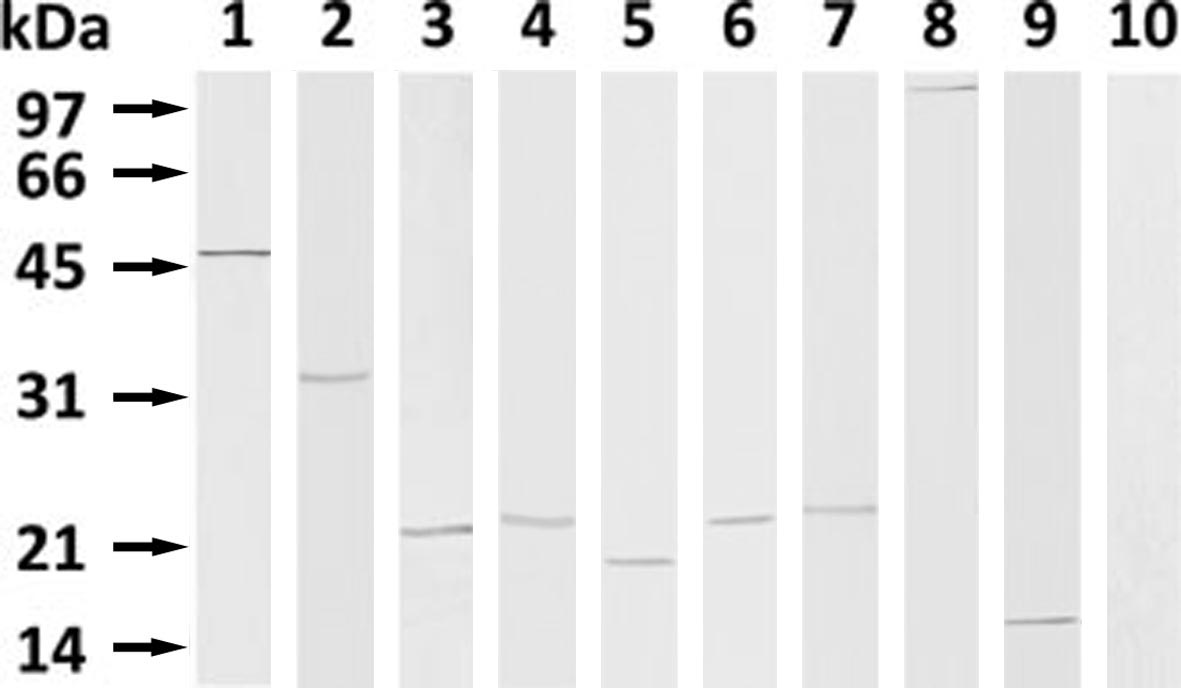

Supplement: Additional file 5: Figure S4. — Western blotting of proteins found in mouse epididymis. Lane 1, clusterin; Lane 2, glyceraldehyde-3-phosphate dehydrogenase; Lane 3, peroxiredoxin-1; Lane 4, superoxide dismutase [Mn]; Lane 5, myosin regulatory light polypeptide 9; Lane 6, glutathione S-transferase P; Lane 7, peroxiredoxin-6; Lane 8, alpha-2 type I collagen; Lane 9, superoxide dismutase [Cu-Zn]; Lane 10, negative control. (JPEG 59 kb) [file 12953_2015_76_MOESM5_ESM.jpeg]
