# Supplementary material for: In-depth Proteomic mapping of mouse (Mus musculus) epididymal constructive basis for sperm maturation
Source: Proteome Sci. 2015 Jul 30;13:20. doi: 10.1186/s12953-015-0076-3 (PMC4518611; doi:10.1186/s12953-015-0076-3)
Supplement: Additional file 3: Table S1. — 625 identified proteins of the mouse epididymis. (DOCX 139 kb) [file 12953_2015_76_MOESM3_ESM.docx]

| Gel spot no. | Swiss-prot AC. | Protein name | Function | Component | Process | MW | pI | Protein score | Unique peptide |
| --- | --- | --- | --- | --- | --- | --- | --- | --- | --- |
| 875 | P84104 | Splicing factor, arginine/serine-rich 3 | protein binding | nucleus | transport | 19317 | 11.46 | 93 | 6 |
| 758 | P19253 | 60S ribosomal protein L13a | structural molecule activity | membrane | regulation of biological process | 23318 | 11.02 | 66 | 4 |
| 678 | Q91WB2 | Probable lipid phosphate phosphatase PPAPDC3 | catalytic activity | membrane | regulation of biological process | 29690 | 10.04 | 61 | 4 |
| 98 | Q01149 | Collagen alpha-2 | structural molecule activity | extracellular matrix | cell adhesion | 91646 | 10 | 103 | 10 |
| 97 | Q01149 | Collagen alpha-2 | structural molecule activity | extracellular matrix | cell adhesion | 91646 | 10 | 172 | 4 |
| 926 | Q30KP0 | Beta-defensin 129 | protein binding | cytoplasm | response to stimulus | 16311 | 9.91 | 139 | 7 |
| 907 | P62983 | Ubiquitin-40S ribosomal protein S27a | hydrolase activity | proteasome | protein metabolic process | 17939.5 | 9.68 | 91 | 5 |
| 908 | P62983 | Ubiquitin-40S ribosomal protein S27a | hydrolase activity | proteasome | protein metabolic process | 17939.5 | 9.68 | 156 | 4 |
| 909 | P62983 | Ubiquitin-40S ribosomal protein S27a | hydrolase activity | proteasome | protein metabolic process | 17939.5 | 9.68 | 156 | 4 |
| 655 | P97931 | Uracil-DNA glycosylase | hydrolase activity | mitochondrion | metabolic process | 33904 | 9.65 | 175 | 5 |
| 656 | P56501 | Mitochondrial uncoupling protein 3 | transporter activity | mitochondrion | response to stimulus | 33888 | 9.61 | 185 | 5 |
| 580 | Q8BK63 | Casein kinase I isoform alpha | transferase activity | cytoplasm | regulation of biological process | 38758 | 9.59 | 62 | 5 |
| 134 | Q8VIJ6 | Splicing factor, proline- and glutamine-rich | protein binding | cytoplasm | regulation of biological process | 75394 | 9.45 | 77 | 4 |
| 853 | P24369 | Peptidyl-prolyl cis-trans isomerase B precursor | isomerase activity | cytoplasm | regulation of biological process | 20205 | 9.42 | 142 | 8 |
| 987 | Q06185 | ATP synthase subunit e, mitochondrial | hydrolase activity | mitochondrion | metabolic process | 8099 | 9.35 | 75 | 6 |
| 46 | Q80XB4 | Nebulin-related-anchoring protein | structural molecule activity | cytoplasm | organelle organization | 195632 | 9.34 | 62 | 8 |
| 742 | Q62446 | Peptidyl-prolyl cis-trans isomerase FKBP3 | isomerase activity | cytoplasm | regulation of biological process | 25001 | 9.29 | 135 | 4 |
| 53 | Q3TX57 | Alpha-2 type I collagen | structural molecule activity | extracellular matrix | cell adhesion | 129556 | 9.27 | 118 | 5 |
| 654 | P49312 | Heterogeneous nuclear ribonucleoprotein L | nucleotide binding | nucleus | regulation of biological process | 34175 | 9.27 | 81 | 8 |
| 238 | P11983 | T-complex protein 1 subunit alpha | nucleotide binding | cytoplasm | protein metabolic process | 59752 | 9.22 | 93 | 4 |
| 949 | Q9JJH1 | Ribonuclease 4 | hydrolase activity | vesicle | metabolic process | 13846 | 9.2 | 68 | 4 |
| 984 | B3GLJ3 | Prostate and testis expressed protein 3 | protein binding | extracellular region | regulation of biological process | 9256 | 9.17 | 62 | 4 |
| 211 | O09118 | Netrin-1 | protein binding | cytoplasm | regulation of biological process | 65355 | 9.09 | 65 | 6 |
| 288 | Q9JJN4 | Succinyl-CoA:3-ketoacid coenzyme A transferase 2A | transferase activity | mitochondrion | metabolic process | 56436.5 | 9.07 | 73 | 4 |
| 122 | Q8BMS1 | Trifunctional enzyme subunit alpha, mitochondrial | catalytic activity | mitochondrion | metabolic process | 78715 | 9.06 | 63 | 4 |
| 911 | O88312 | Anterior gradient protein 2 homolog | protein binding | extracellular region | multicellular organismal process | 17863 | 9.06 | 74 | 5 |
| 910 | O88312 | Anterior gradient protein 2 homolog | protein binding | extracellular region | multicellular organismal process | 17863 | 9.06 | 175 | 5 |
| 657 | Q08091 | Calponin-1 | protein binding | cytoskeleton | organelle organization | 33334 | 9.05 | 69 | 6 |
| 734 | P13745 | Glutathione S-transferase A1 | transferase activity | cytoplasm | metabolic process | 25591 | 9.04 | 148 | 5 |
| 93 | P28481 | Collagen alpha-1(II) chain | structural molecule activity | extracellular matrix | cell adhesion | 96123 | 9.02 | 125 | 4 |
| 92 | P28481 | Collagen alpha-1(II) chain | structural molecule activity | extracellular matrix | cell adhesion | 96123 | 9.02 | 144 | 5 |
| 957 | P21460 | Cystatin-C | enzyme regulator activity | extracellular region | regulation of biological process | 13397 | 8.99 | 90 | 4 |
| 622 | O88569 | Heterogeneous nuclear ribonucleoproteins A2/B1 | nucleotide binding | nucleus | regulation of biological process | 37379 | 8.97 | 60 | 4 |
| 621 | O88569 | Heterogeneous nuclear ribonucleoproteins A2/B1 | nucleotide binding | nucleus | regulation of biological process | 37379 | 8.97 | 115 | 8 |
| 203 | B2RSM1 | NADPH oxidase 4 | antioxidant activity | cytoplasm | metabolic process | 66519 | 8.96 | 77 | 4 |
| 841 | P97314 | Cysteine and glycine-rich protein 2 | enzyme regulator activity | extracellular region | regulation of biological process | 20911 | 8.95 | 82 | 6 |
| 974 | Q09098 | Prostate and testis expressed protein 4 | protein binding | extracellular region | cell communication | 11058.1 | 8.93 | 86 | 4 |
| 679 | Q9CR68 | Cytochrome b-c1 complex subunit Rieske, mitochondrial | transporter activity | mitochondrion | transport | 29634 | 8.91 | 128 | 4 |
| 850 | P97315 | Cysteine and glycine-rich protein 1 | enzyme regulator activity | extracellular region | regulation of biological process | 20569 | 8.9 | 82 | 6 |
| 424 | P54071 | Isocitrate dehydrogenase [NADP] | oxidoreductase activity | mitochondrion | carbohydrate metabolic process | 50906.18 | 8.88 | 86 | 4 |
| 736 | P62827 | GTP-binding nuclear protein Ran | nucleotide binding | mitochondrion | nucleotide metabolic process | 25279 | 8.88 | 62 | 5 |
| 737 | Q9WTP7 | GTP:AMP phosphotransferase AK3 | nucleotide binding | mitochondrion | nucleotide metabolic process | 25279 | 8.88 | 80 | 6 |
| 783 | P37804 | Transgelin | protein binding | vesicle | regulation of biological process | 22430 | 8.86 | 130 | 12 |
| 781 | P37804 | Transgelin | protein binding | vesicle | regulation of biological process | 22430 | 8.86 | 131 | 12 |
| 668 | Q4KUS1 | WAP four-disulfide core domain protein 8 | enzyme regulator activity | membrane | regulation of biological process | 31256 | 8.85 | 90 | 5 |
| 627 | Q8C8B0 | ALX homeobox protein 1 | protein binding | nucleus | regulation of biological process | 36908 | 8.8 | 68 | 6 |
| 748 | P09671 | Superoxide dismutase [Mn], mitochondrial | antioxidant activity | mitochondrion | metabolic process | 24602 | 8.8 | 87 | 4 |
| 746 | P09671 | Superoxide dismutase [Mn], mitochondrial | antioxidant activity | mitochondrion | metabolic process | 24602 | 8.8 | 109 | 5 |
| 48 | P08122 | Collagen alpha-2(IV) chain | structural molecule activity | extracellular matrix | cell adhesion | 167324 | 8.75 | 87 | 4 |
| 362 | Q3U6K5 | spermatogenesis associated 6 | binding | extracellular region | multicellular organismal process | 54213 | 8.71 | 75 | 5 |
| 215 | Q99MQ5 | Collagen alpha-1 chain precursor | structural molecule activity | extracellular matrix | cell adhesion | 65336 | 8.7 | 70 | 6 |
| 214 | Q99MQ5 | Collagen alpha-1 chain precursor | structural molecule activity | extracellular matrix | cell adhesion | 65336 | 8.7 | 82 | 9 |
| 512 | O54799 | Neuromedin-B receptor | binding | cytoplasm | cell communication | 43594 | 8.63 | 60 | 5 |
| 628 | Q01339 | Beta-2-glycoprotein 1 precursor | protein binding | extracellular region | regulation of biological process | 36622 | 8.62 | 64 | 5 |
| 958 | Q9DA01 | Eppin | enzyme regulator activity | cytoplasm | regulation of biological process | 13230 | 8.59 | 62 | 5 |
| 228 | Q8CHT0 | Delta-1-pyrroline-5-carboxylate dehydrogenase, mitochondrial | oxidoreductase activity | mitochondrion | metabolic process | 62228 | 8.58 | 116 | 9 |
| 225 | Q8CHT0 | Delta-1-pyrroline-5-carboxylate dehydrogenase, mitochondrial | oxidoreductase activity | mitochondrion | metabolic process | 62228 | 8.58 | 127 | 7 |
| 224 | Q8CHT0 | Delta-1-pyrroline-5-carboxylate dehydrogenase, mitochondrial | oxidoreductase activity | mitochondrion | metabolic process | 62228 | 8.58 | 140 | 9 |
| 226 | Q8CHT0 | Delta-1-pyrroline-5-carboxylate dehydrogenase, mitochondrial | oxidoreductase activity | mitochondrion | metabolic process | 62228 | 8.58 | 159 | 5 |
| 227 | Q8CHT0 | Delta-1-pyrroline-5-carboxylate dehydrogenase, mitochondrial | oxidoreductase activity | mitochondrion | metabolic process | 62228 | 8.58 | 181 | 4 |
| 720 | O08756 | 3-hydroxyacyl-CoA dehydrogenase type-2 | oxidoreductase activity | mitochondrion | metabolic process | 27270 | 8.56 | 118 | 10 |
| 468 | Q9JHI5 | Isovaleryl-CoA dehydrogenase | oxidoreductase activity | mitochondrion | metabolic process | 46295.9 | 8.52 | 93 | 5 |
| 717 | P16110 | Galectin-3 | protein binding | membrane | cell adhesion | 27366 | 8.5 | 60 | 4 |
| 815 | O88425 | Nucleoside diphosphate kinase 6 | nucleotide binding | cytoplasm | nucleotide metabolic process | 21777 | 8.48 | 78 | 5 |
| 642 | P16858 | Glyceraldehyde-3-phosphate dehydrogenase | oxidoreductase activity | cytoplasm | carbohydrate metabolic process | 35787.2 | 8.44 | 133 | 5 |
| 641 | P16858 | Glyceraldehyde-3-phosphate dehydrogenase | oxidoreductase activity | cytoplasm | carbohydrate metabolic process | 35787.2 | 8.44 | 135 | 4 |
| 793 | Q9WVA4 | Transgelin-2 | protein binding | vesicle | regulation of biological process | 22250 | 8.44 | 112 | 4 |
| 792 | Q9WVA4 | Transgelin-2 | protein binding | vesicle | regulation of biological process | 22250 | 8.44 | 144 | 4 |
| 577 | P00329 | Alcohol dehydrogenase 1 | oxidoreductase activity | mitochondrion | metabolic process | 39614 | 8.43 | 138 | 8 |
| 640 | Q548Z6 | L-lactate dehydrogenase A chain | catalytic activity | cytoplasm | carbohydrate metabolic process | 35889 | 8.43 | 133 | 4 |
| 459 | P16460 | Argininosuccinate synthase | nucleotide binding | vesicle | cell adhesion | 46840 | 8.36 | 63 | 4 |
| 458 | P16460 | Argininosuccinate synthase | nucleotide binding | vesicle | cell adhesion | 46840 | 8.36 | 104 | 4 |
| 425 | Q8K0E8 | Fibrinogen beta chain precursor | structural molecule activity | membrane | cell adhesion | 50786 | 8.35 | 62 | 6 |
| 570 | Q8BWT1 | 3-ketoacyl-CoA thiolase, mitochondrial | catalytic activity | vesicle | metabolic process | 41803 | 8.33 | 138 | 4 |
| 569 | Q8BWT1 | 3-ketoacyl-CoA thiolase, mitochondrial | catalytic activity | vesicle | metabolic process | 41803 | 8.33 | 139 | 4 |
| 509 | P47809 | Mitogen-activated protein kinase kinase kinase kinase 4 | transferase activity | cytoplasm | cell communication | 43954 | 8.31 | 62 | 6 |
| 579 | P05064 | Fructose-bisphosphate aldolase A | catalytic activity | cytoplasm | carbohydrate metabolic process | 39331.3 | 8.31 | 104 | 7 |
| 423 | O55222 | Integrin-linked protein kinase | transferase activity | cytoplasm | regulation of biological process | 51339 | 8.3 | 61 | 6 |
| 244 | Q9EQ20 | Methylmalonate-semialdehyde dehydrogenase [acylating] | oxidoreductase activity | mitochondrion | metabolic process | 57878.5 | 8.29 | 79 | 6 |
| 802 | P35700 | Peroxiredoxin-1 | antioxidant activity | cytoplasm | metabolic process | 22176 | 8.26 | 61 | 4 |
| 801 | P35700 | Peroxiredoxin-1 | antioxidant activity | cytoplasm | metabolic process | 22176 | 8.26 | 70 | 5 |
| 800 | P35700 | Peroxiredoxin-1 | antioxidant activity | cytoplasm | metabolic process | 22176 | 8.26 | 75 | 6 |
| 799 | P35700 | Peroxiredoxin-1 | antioxidant activity | cytoplasm | metabolic process | 22176 | 8.26 | 79 | 5 |
| 798 | P35700 | Peroxiredoxin-1 | antioxidant activity | cytoplasm | metabolic process | 22176 | 8.26 | 80 | 6 |
| 797 | P35700 | Peroxiredoxin-1 | antioxidant activity | cytoplasm | metabolic process | 22176 | 8.26 | 98 | 6 |
| 794 | P35700 | Peroxiredoxin-1 | antioxidant activity | cytoplasm | metabolic process | 22176 | 8.26 | 117 | 8 |
| 944 | P32766 | Cystatin-8 | enzyme regulator activity | extracellular region | regulation of biological process | 14187 | 8.26 | 61 | 5 |
| 241 | Q564F4 | T-complex protein 1 subunit delta | nucleotide binding | cytoplasm | protein metabolic process | 58066 | 8.24 | 89 | 5 |
| 890 | P18760 | Cofilin-1 | structural molecule activity | cytoplasm | multicellular organismal process | 18547.7 | 8.22 | 60 | 4 |
| 888 | P18760 | Cofilin-1 | structural molecule activity | cytoplasm | multicellular organismal process | 18547.7 | 8.22 | 63 | 4 |
| 887 | P18760 | Cofilin-1 | structural molecule activity | cytoplasm | multicellular organismal process | 18547.7 | 8.22 | 88 | 5 |
| 884 | P18760 | Cofilin-1 | structural molecule activity | cytoplasm | multicellular organismal process | 18547.7 | 8.22 | 101 | 6 |
| 882 | P18760 | Cofilin-1 | structural molecule activity | cytoplasm | multicellular organismal process | 18547.7 | 8.22 | 105 | 6 |
| 892 | Q9R0P5 | Destrin | protein binding | cytoplasm | organelle organization | 18390 | 8.19 | 99 | 7 |
| 976 | Q64433 | 10 kDa heat shock protein, mitochondrial | antioxidant activity | cytoplasm | regulation of biological process | 10824 | 8.18 | 62 | 4 |
| 204 | Q9JLN6 | Disintegrin and metalloproteinase domain-containing protein 28 | structural molecule activity | membrane | protein metabolic process | 66483 | 8.17 | 104 | 8 |
| 934 | Q5GAM7 | Ribonuclease-like protein 13 | nucleotide binding | extracellular region | regulation of biological process | 15191 | 8.14 | 79 | 4 |
| 108 | Q99KI0 | Aconitate hydratase, mitochondrial | lyase activity | mitochondrion | metabolic process | 85410 | 8.08 | 78 | 4 |
| 111 | Q99KI0 | Aconitate hydratase, mitochondrial | lyase activity | mitochondrion | metabolic process | 85410 | 8.08 | 83 | 7 |
| 110 | Q99KI0 | Aconitate hydratase, mitochondrial | lyase activity | mitochondrion | metabolic process | 85410 | 8.08 | 84 | 6 |
| 114 | Q99KI0 | Aconitate hydratase, mitochondrial | lyase activity | mitochondrion | metabolic process | 85410 | 8.08 | 98 | 5 |
| 116 | Q99KI0 | Aconitate hydratase, mitochondrial | lyase activity | mitochondrion | metabolic process | 85410 | 8.08 | 141 | 4 |
| 969 | P26883 | Peptidyl-prolyl cis-trans isomerase FKBP1A | isomerase activity | cytoplasm | regulation of biological process | 11784 | 8.08 | 125 | 9 |
| 123 | P97857 | A disintegrin and metalloproteinase with thrombospondin motifs 1 | structural molecule activity | cytoplasm | multicellular organismal process | 78514 | 8.07 | 133 | 4 |
| 483 | O35490 | Betaine--homocysteine S-methyltransferase 1 | protein binding | cytoplasm | regulation of biological process | 44860 | 8.07 | 70 | 6 |
| 735 | P19639 | Glutathione S-transferase Mu 3 | transferase activity | cytoplasm | metabolic process | 25553 | 8.05 | 123 | 4 |
| 484 | P09411 | Phosphoglycerate kinase 1 | transferase activity | cytoplasm | carbohydrate metabolic process | 44550 | 8.02 | 117 | 4 |
| 486 | P09411 | Phosphoglycerate kinase 1 | transferase activity | cytoplasm | carbohydrate metabolic process | 44550 | 8.02 | 117 | 5 |
| 487 | P09411 | Phosphoglycerate kinase 1 | transferase activity | cytoplasm | carbohydrate metabolic process | 44550 | 8.02 | 117 | 5 |
| 359 | O08749 | Dihydrolipoyl dehydrogenase, mitochondrial | oxidoreductase activity | mitochondrion | regulation of biological process | 54238.2 | 7.99 | 79 | 7 |
| 358 | O08749 | Dihydrolipoyl dehydrogenase, mitochondrial | oxidoreductase activity | mitochondrion | regulation of biological process | 54238.2 | 7.99 | 86 | 5 |
| 357 | O08749 | Dihydrolipoyl dehydrogenase, mitochondrial | oxidoreductase activity | mitochondrion | regulation of biological process | 54238.2 | 7.99 | 109 | 4 |
| 637 | O70479 | BTB/POZ domain-containing protein 10 | protein binding | cytoplasm | regulation of biological process | 36111 | 7.99 | 61 | 5 |
| 961 | F6ULY1 | WAP four-disulfide core domain protein 6 | enzyme regulator activity | membrane | regulation of biological process | 13093 | 7.99 | 62 | 4 |
| 337 | P24549 | Retinal dehydrogenase 1 | oxidoreductase activity | cytoplasm | metabolic process | 54446.9 | 7.89 | 62 | 5 |
| 336 | P24549 | Retinal dehydrogenase 1 | oxidoreductase activity | cytoplasm | metabolic process | 54446.9 | 7.89 | 92 | 6 |
| 334 | P24549 | Retinal dehydrogenase 1 | oxidoreductase activity | cytoplasm | metabolic process | 54446.9 | 7.89 | 101 | 4 |
| 346 | P24549 | Retinal dehydrogenase 1 | oxidoreductase activity | cytoplasm | metabolic process | 54446.9 | 7.89 | 124 | 4 |
| 348 | P24549 | Retinal dehydrogenase 1 | oxidoreductase activity | cytoplasm | metabolic process | 54446.9 | 7.89 | 124 | 4 |
| 349 | P24549 | Retinal dehydrogenase 1 | oxidoreductase activity | cytoplasm | metabolic process | 54446.9 | 7.89 | 124 | 4 |
| 345 | P24549 | Retinal dehydrogenase 1 | oxidoreductase activity | cytoplasm | metabolic process | 54446.9 | 7.89 | 125 | 4 |
| 342 | P24549 | Retinal dehydrogenase 1 | oxidoreductase activity | cytoplasm | metabolic process | 54446.9 | 7.89 | 154 | 5 |
| 340 | P24549 | Retinal dehydrogenase 1 | oxidoreductase activity | cytoplasm | metabolic process | 54446.9 | 7.89 | 157 | 4 |
| 658 | P52196 | Thiosulfate sulfurtransferase | transferase activity | vesicle | metabolic process | 33313 | 7.82 | 84 | 7 |
| 960 | Q9D269 | Cystatin-11 | enzyme regulator activity | extracellular region | regulation of biological process | 13148 | 7.82 | 92 | 4 |
| 699 | Q8BH95 | Enoyl-CoA hydratase, mitochondrial precursor | catalytic activity | vesicle | metabolic process | 28474 | 7.78 | 95 | 8 |
| 698 | Q8BH95 | Enoyl-CoA hydratase, mitochondrial precursor | catalytic activity | vesicle | metabolic process | 28474 | 7.78 | 123 | 9 |
| 902 | P17742 | Peptidyl-prolyl cis-trans isomerase A | isomerase activity | cytoplasm | regulation of biological process | 17959.8 | 7.74 | 71 | 6 |
| 901 | P17742 | Peptidyl-prolyl cis-trans isomerase A | isomerase activity | cytoplasm | regulation of biological process | 17959.8 | 7.74 | 82 | 4 |
| 899 | P17742 | Peptidyl-prolyl cis-trans isomerase A | isomerase activity | cytoplasm | regulation of biological process | 17959.8 | 7.74 | 99 | 9 |
| 898 | P17742 | Peptidyl-prolyl cis-trans isomerase A | isomerase activity | cytoplasm | regulation of biological process | 17959.8 | 7.74 | 109 | 7 |
| 896 | P17742 | Peptidyl-prolyl cis-trans isomerase A | isomerase activity | cytoplasm | regulation of biological process | 17959.8 | 7.74 | 119 | 10 |
| 905 | P17742 | Peptidyl-prolyl cis-trans isomerase A | isomerase activity | cytoplasm | regulation of biological process | 17959.8 | 7.74 | 123 | 4 |
| 904 | P17742 | Peptidyl-prolyl cis-trans isomerase A | isomerase activity | cytoplasm | regulation of biological process | 17959.8 | 7.74 | 154 | 5 |
| 895 | P17742 | Peptidyl-prolyl cis-trans isomerase A | isomerase activity | cytoplasm | regulation of biological process | 17959.8 | 7.74 | 164 | 5 |
| 893 | P17742 | Peptidyl-prolyl cis-trans isomerase A | isomerase activity | cytoplasm | regulation of biological process | 17959.8 | 7.74 | 165 | 5 |
| 894 | P17742 | Peptidyl-prolyl cis-trans isomerase A | isomerase activity | cytoplasm | regulation of biological process | 17959.8 | 7.74 | 165 | 4 |
| 236 | P24270 | Catalase | antioxidant activity | cytoplasm | multicellular organismal process | 60013 | 7.72 | 64 | 7 |
| 235 | P24270 | Catalase | antioxidant activity | cytoplasm | multicellular organismal process | 60013 | 7.72 | 104 | 4 |
| 234 | P24270 | Catalase | antioxidant activity | cytoplasm | multicellular organismal process | 60013 | 7.72 | 108 | 4 |
| 237 | P24270 | Catalase | antioxidant activity | cytoplasm | multicellular organismal process | 60013 | 7.72 | 124 | 4 |
| 975 | Q3UW31 | Prostate and testis expressed protein 2 | protein binding | extracellular region | regulation of biological process | 10906 | 7.72 | 60 | 6 |
| 229 | Q06649 | SH3 domain-binding protein 2 | protein binding | nucleus | regulation of biological process | 62169 | 7.71 | 63 | 5 |
| 726 | P10649 | Glutathione S-transferase Mu 1 | transferase activity | cytoplasm | metabolic process | 25953.1 | 7.71 | 76 | 4 |
| 724 | P10649 | Glutathione S-transferase Mu 1 | transferase activity | cytoplasm | metabolic process | 25953.1 | 7.71 | 153 | 4 |
| 920 | P99029 | Peroxiredoxin-5 | antioxidant activity | cytoplasm | metabolic process | 17004 | 7.7 | 116 | 7 |
| 514 | P45952 | Medium-chain specific acyl-CoA dehydrogenase, mitochondrial | protein binding | vesicle | metabolic process | 43565 | 7.69 | 121 | 4 |
| 753 | P19157 | Glutathione S-transferase P 1 | transferase activity | cytoplasm | metabolic process | 23594.1 | 7.68 | 110 | 5 |
| 754 | P19157 | Glutathione S-transferase P 1 | transferase activity | cytoplasm | metabolic process | 23594.1 | 7.68 | 110 | 4 |
| 756 | P19157 | Glutathione S-transferase P 1 | transferase activity | cytoplasm | metabolic process | 23594.1 | 7.68 | 110 | 4 |
| 182 | Q9Z2C5 | Myotubularin | structural molecule activity | cytoplasm | regulation of biological process | 69515 | 7.65 | 64 | 5 |
| 183 | Q9Z2C5 | Myotubularin | structural molecule activity | cytoplasm | regulation of biological process | 69515 | 7.65 | 64 | 5 |
| 178 | Q9Z2C5 | Myotubularin | structural molecule activity | cytoplasm | regulation of biological process | 69515 | 7.65 | 75 | 6 |
| 179 | Q9Z2C5 | Myotubularin | structural molecule activity | cytoplasm | regulation of biological process | 69515 | 7.65 | 75 | 6 |
| 180 | Q9Z2C5 | Myotubularin | structural molecule activity | cytoplasm | regulation of biological process | 69515 | 7.65 | 75 | 6 |
| 181 | Q9Z2C5 | Myotubularin | structural molecule activity | cytoplasm | regulation of biological process | 69515 | 7.65 | 75 | 6 |
| 177 | Q9Z2C5 | Myotubularin | structural molecule activity | cytoplasm | regulation of biological process | 69515 | 7.65 | 77 | 7 |
| 184 | Q9Z2C5 | Myotubularin | structural molecule activity | cytoplasm | regulation of biological process | 69515 | 7.65 | 110 | 5 |
| 185 | Q9Z2C5 | Myotubularin | structural molecule activity | cytoplasm | regulation of biological process | 69515 | 7.65 | 146 | 4 |
| 187 | Q9Z2C5 | Myotubularin | structural molecule activity | cytoplasm | regulation of biological process | 69515 | 7.65 | 146 | 4 |
| 321 | Q9CPY7 | Cytosol aminopeptidase | hydrolase activity | cytoplasm | protein metabolic process | 56105.8 | 7.62 | 110 | 4 |
| 315 | Q9CPY7 | Cytosol aminopeptidase | hydrolase activity | cytoplasm | protein metabolic process | 56105.8 | 7.62 | 119 | 5 |
| 316 | Q9CPY7 | Cytosol aminopeptidase | hydrolase activity | cytoplasm | protein metabolic process | 56105.8 | 7.62 | 119 | 4 |
| 323 | Q9CPY7 | aminopeptidase | hydrolase activity | cytoplasm | protein metabolic process | 56105 | 7.61 | 66 | 8 |
| 649 | P68040 | Guanine nucleotide-binding protein subunit beta 2-like 1 | nucleotide binding | mitochondrion | nucleotide metabolic process | 35054 | 7.6 | 60 | 5 |
| 925 | Q9Z0J0 | Epididymal secretory protein E1 | binding | cytoplasm | metabolic process | 16431.5 | 7.59 | 69 | 5 |
| 647 | Q91Z53 | Glyoxylate reductase/hydroxypyruvate reductase | nucleotide binding | cytoplasm | metabolic process | 35306 | 7.57 | 89 | 7 |
| 287 | P47738 | Aldehyde dehydrogenase, mitochondrial | oxidoreductase activity | mitochondrion | metabolic process | 56501.8 | 7.53 | 88 | 4 |
| 286 | P47738 | Aldehyde dehydrogenase, mitochondrial | oxidoreductase activity | mitochondrion | metabolic process | 56501.8 | 7.53 | 108 | 6 |
| 284 | P47738 | Aldehyde dehydrogenase, mitochondrial | oxidoreductase activity | mitochondrion | metabolic process | 56501.8 | 7.53 | 119 | 4 |
| 364 | P97384 | Annexin A11 | structural molecule activity | vesicle | response to stimulus | 54044 | 7.53 | 92 | 9 |
| 591 | P07356 | Annexin A2 | structural molecule activity | vesicle | response to stimulus | 38520 | 7.53 | 61 | 6 |
| 587 | P07356 | Annexin A2 | structural molecule activity | vesicle | response to stimulus | 38520 | 7.53 | 77 | 6 |
| 600 | P07356 | Annexin A2 | structural molecule activity | vesicle | response to stimulus | 38520 | 7.53 | 114 | 4 |
| 599 | P07356 | Annexin A2 | structural molecule activity | vesicle | response to stimulus | 38520 | 7.53 | 118 | 4 |
| 597 | P07356 | Annexin A2 | structural molecule activity | vesicle | response to stimulus | 38520 | 7.53 | 133 | 5 |
| 594 | P07356 | Annexin A2 | structural molecule activity | vesicle | response to stimulus | 38520 | 7.53 | 135 | 5 |
| 585 | P07356 | Annexin A2 | structural molecule activity | vesicle | response to stimulus | 38520 | 7.53 | 139 | 6 |
| 586 | P07356 | Annexin A2 | structural molecule activity | vesicle | response to stimulus | 38520 | 7.53 | 139 | 4 |
| 607 | O09005 | Sphingolipid delta(4)-desaturase DES1 | oxidoreductase activity | mitochondrion | regulation of biological process | 38085 | 7.44 | 70 | 5 |
| 664 | Q60930 | Voltage-dependent anion-selective channel protein 2 | transporter activity | vesicle | transport | 31712 | 7.44 | 94 | 7 |
| 246 | P52480 | Pyruvate kinase PKM | catalytic activity | cytoplasm | carbohydrate metabolic process | 57676 | 7.42 | 149 | 14 |
| 247 | P52480 | Pyruvate kinase PKM | catalytic activity | cytoplasm | carbohydrate metabolic process | 57676 | 7.42 | 157 | 5 |
| 752 | Q9D172 | ES1 protein homolog, mitochondrial precursor | protein binding | mitochondrion | regulation of biological process | 23919 | 7.31 | 89 | 7 |
| 88 | P28271 | Cytoplasmic aconitate hydratase | oxidoreductase activity | mitochondrion | metabolic process | 98063 | 7.23 | 89 | 5 |
| 89 | P28271 | Cytoplasmic aconitate hydratase | oxidoreductase activity | mitochondrion | metabolic process | 98063 | 7.23 | 89 | 4 |
| 90 | P28271 | Cytoplasmic aconitate hydratase | oxidoreductase activity | mitochondrion | metabolic process | 98063 | 7.23 | 157 | 12 |
| 201 | P40142 | Transketolase | transferase activity | nucleus | metabolic process | 67587.6 | 7.23 | 65 | 4 |
| 200 | P40142 | Transketolase | transferase activity | nucleus | metabolic process | 67587.6 | 7.23 | 93 | 5 |
| 240 | P32020 | Non-specific lipid-transfer protein | catalytic activity | cytoplasm | metabolic process | 59087 | 7.16 | 97 | 10 |
| 651 | P70190 | F-actin-capping protein subunit alpha-3 | structural molecule activity | membrane | multicellular organismal process | 34929 | 7.16 | 129 | 7 |
| 583 | P10107 | Annexin A1 | structural molecule activity | vesicle | response to stimulus | 38578 | 7.15 | 119 | 9 |
| 704 | P20108 | Thioredoxin-dependent peroxide reductase | antioxidant activity | cytoplasm | metabolic process | 28109.4 | 7.15 | 71 | 5 |
| 703 | P20108 | Thioredoxin-dependent peroxide reductase | antioxidant activity | cytoplasm | metabolic process | 28109.4 | 7.15 | 88 | 4 |
| 728 | P49722 | Proteasome subunit alpha type 2 | hydrolase activity | proteasome | protein metabolic process | 25779 | 7.12 | 68 | 6 |
| 578 | P28474 | Alcohol dehydrogenase class-3 | oxidoreductase activity | mitochondrion | metabolic process | 39391 | 7.11 | 74 | 5 |
| 492 | P62334 | 26S protease regulatory subunit 10B | hydrolase activity | proteasome | protein metabolic process | 44173.1 | 7.1 | 109 | 5 |
| 938 | Q5GAM8 | Ribonuclease-like protein 12 | nucleotide binding | extracellular region | regulation of biological process | 14634 | 7.09 | 60 | 5 |
| 519 | P30678 | Guanine nucleotide-binding protein alpha-15 subunit | nucleotide binding | mitochondrion | nucleotide metabolic process | 43508 | 7.07 | 62 | 7 |
| 940 | Q6JVL5 | Epididymal-specific lipocalin-12 | transporter activity | extracellular region | transport | 14486 | 7.04 | 111 | 5 |
| 741 | Q9WUR9 | Adenylate kinase 4, mitochondrial | nucleotide binding | vesicle | nucleotide metabolic process | 25046 | 7.02 | 65 | 5 |
| 414 | Q9D0K2 | 3-ketoacid-coenzyme A transferase 1 | catalytic activity | vesicle | metabolic process | 51843 | 7.01 | 88 | 9 |
| 418 | Q9D0K2 | 3-ketoacid-coenzyme A transferase 0 | catalytic activity | vesicle | metabolic process | 51843 | 7.01 | 114 | 9 |
| 262 | Q921X9 | Protein disulfide-isomerase A5 | oxidoreductase activity | cytoplasm | regulation of biological process | 56993 | 6.98 | 99 | 4 |
| 261 | Q921X9 | Protein disulfide-isomerase A5 | oxidoreductase activity | cytoplasm | regulation of biological process | 56993 | 6.98 | 109 | 4 |
| 263 | Q921X9 | Protein disulfide-isomerase A5 | oxidoreductase activity | cytoplasm | regulation of biological process | 56993 | 6.98 | 139 | 4 |
| 683 | P16015 | Carbonic anhydrase 3 | ion binding | cytoplasm | response to stimulus | 29216 | 6.97 | 110 | 7 |
| 682 | P16015 | Carbonic anhydrase 3 | ion binding | cytoplasm | response to stimulus | 29216 | 6.97 | 165 | 10 |
| 916 | Q01768 | Nucleoside diphosphate kinase B | nucleotide binding | cytoplasm | nucleotide metabolic process | 17351.9 | 6.97 | 76 | 5 |
| 915 | Q01768 | Nucleoside diphosphate kinase B | nucleotide binding | cytoplasm | nucleotide metabolic process | 17351.9 | 6.97 | 83 | 6 |
| 131 | Q921I1 | Serotransferrin | protein binding | cytoplasm | regulation of biological process | 76673.7 | 6.94 | 77 | 5 |
| 132 | Q921I1 | Serotransferrin | protein binding | cytoplasm | regulation of biological process | 76673.7 | 6.94 | 97 | 4 |
| 133 | Q921I1 | Serotransferrin | protein binding | cytoplasm | regulation of biological process | 76673.7 | 6.94 | 98 | 5 |
| 328 | P24547 | Inosine-5'-monophosphate dehydrogenase 2 | catalytic activity | cytoplasm | nucleotide metabolic process | 55648 | 6.92 | 98 | 8 |
| 466 | P35505 | Fumarylacetoacetase | hydrolase activity | vesicle | metabolic process | 46416 | 6.92 | 157 | 4 |
| 42 | Q91V24 | ATP-binding cassette sub-family A member 7 | hydrolase activity | mitochondrion | metabolic process | 236733 | 6.91 | 64 | 5 |
| 43 | Q91V24 | ATP-binding cassette sub-family A member 7 | hydrolase activity | mitochondrion | metabolic process | 236733 | 6.91 | 159 | 5 |
| 422 | Q9WVS6 | E3 ubiquitin-protein ligase parkin | protein binding | cytoplasm | protein metabolic process | 51583 | 6.9 | 144 | 4 |
| 722 | P17751 | Triosephosphate isomerase | catalytic activity | vesicle | carbohydrate metabolic process | 27038 | 6.9 | 66 | 5 |
| 723 | P17751 | Triosephosphate isomerase | catalytic activity | vesicle | carbohydrate metabolic process | 27038 | 6.9 | 154 | 5 |
| 732 | P15626 | Glutathione S-transferase Mu 2 | transferase activity | cytoplasm | metabolic process | 25700 | 6.9 | 90 | 8 |
| 719 | O09131 | Glutathione transferase omega-1 | transferase activity | cytoplasm | metabolic process | 27348 | 6.89 | 60 | 5 |
| 718 | O09131 | Glutathione transferase omega-1 | transferase activity | cytoplasm | metabolic process | 27348 | 6.89 | 65 | 5 |
| 631 | Q9JII6 | Alcohol dehydrogenase [NADP+] | oxidoreductase activity | cytoplasm | carbohydrate metabolic process | 36432 | 6.87 | 71 | 7 |
| 632 | Q9JII6 | Alcohol dehydrogenase [NADP+] | oxidoreductase activity | cytoplasm | carbohydrate metabolic process | 36432 | 6.87 | 124 | 7 |
| 652 | Q9DCT1 | 1,5-anhydro-D-fructose reductase | oxidoreductase activity | cytoplasm | oxidation-reduction process | 34460.7 | 6.87 | 98 | 4 |
| 153 | Q8VCD5 | Mediator of RNA polymerase II transcription subunit 17 | protein binding | nucleus | cell communication | 72416 | 6.86 | 65 | 7 |
| 918 | P15532 | Nucleoside diphosphate kinase A | nucleotide binding | cytoplasm | nucleotide metabolic process | 17196.8 | 6.84 | 86 | 5 |
| 917 | P15532 | Nucleoside diphosphate kinase A | nucleotide binding | cytoplasm | nucleotide metabolic process | 17196.8 | 6.84 | 95 | 7 |
| 729 | Q91X52 | L-xylulose reductase | oxidoreductase activity | vesicle | carbohydrate metabolic process | 25729 | 6.83 | 64 | 4 |
| 558 | Q9JJ06 | Glycoprotein-N-acetylgalactosamine 3-beta-galactosyltransferase 1 | transferase activity | membrane | multicellular organismal process | 42145 | 6.78 | 65 | 5 |
| 639 | P21300 | Aldose reductase-related protein 1 | oxidoreductase activity | mitochondrion | metabolic process | 35965.8 | 6.77 | 120 | 4 |
| 174 | Q5F259 | Ankyrin repeat domain-containing protein 13B | protein binding | membrane | regulation of biological process | 70329 | 6.76 | 61 | 6 |
| 283 | P16277 | Tyrosine-protein kinase BLK | transferase activity | cytoplasm | cell communication | 56507 | 6.76 | 61 | 5 |
| 854 | P23927 | Alpha crystallin B chain | structural molecule activity | cytoplasm | regulation of biological process | 20056 | 6.76 | 65 | 4 |
| 619 | P13707 | Glycerol-3-phosphate dehydrogenase | oxidoreductase activity | cytoplasm | carbohydrate metabolic process | 37548 | 6.75 | 117 | 10 |
| 692 | Q9DBJ1 | Phosphoglycerate mutase 1 | catalytic activity | cytoplasm | carbohydrate metabolic process | 28682 | 6.75 | 102 | 8 |
| 465 | O88844 | Isocitrate dehydrogenase [NADP] cytoplasmic | oxidoreductase activity | cytoplasm | carbohydrate metabolic process | 46674.5 | 6.73 | 125 | 9 |
| 461 | O88844 | Isocitrate dehydrogenase [NADP] cytoplasmic | oxidoreductase activity | cytoplasm | carbohydrate metabolic process | 46674.5 | 6.73 | 149 | 12 |
| 488 | Q80UP4 | Non-metastatic cells 7 | nucleotide binding | cytoskeleton | nucleotide metabolic process | 44493 | 6.72 | 71 | 5 |
| 326 | P26443 | Glutamate dehydrogenase 1 | nucleotide binding | cytoplasm | cell communication | 55877 | 6.71 | 116 | 12 |
| 644 | P45376 | Aldose reductase | oxidoreductase activity | cytoplasm | metabolic process | 35695.4 | 6.71 | 90 | 4 |
| 260 | Q9ERE3 | Serine/threonine-protein kinase Sgk3 | transferase activity | cytoplasm | cell communication | 57109 | 6.69 | 64 | 14 |
| 469 | P97807 | Fumarate hydratase | catalytic activity | cytoplasm | multicellular organismal process | 46015 | 6.69 | 62 | 5 |
| 626 | Q54A37 | Aldo-keto reductase family 1 member C13 | oxidoreductase activity | cytoplasm | metabolic process | 37057.5 | 6.67 | 119 | 4 |
| 675 | O08807 | Peroxiredoxin-4 | antioxidant activity | cytoplasm | metabolic process | 31052 | 6.67 | 73 | 4 |
| 50 | P84309 | Adenylate cyclase type 5 | nucleotide binding | membrane | cell communication | 139032 | 6.65 | 141 | 14 |
| 563 | P15105 | Glutamine synthetase | catalytic activity | cytoplasm | regulation of biological process | 42092.3 | 6.64 | 92 | 6 |
| 561 | P15105 | Glutamine synthetase | catalytic activity | cytoplasm | regulation of biological process | 42092.3 | 6.64 | 137 | 11 |
| 983 | P43024 | Cytochrome c oxidase subunit 6A1 | transporter activity | mitochondrion | transport | 9744.8 | 6.64 | 75 | 6 |
| 482 | P09041 | Phosphoglycerate kinase 2 | transferase activity | cytoplasm | carbohydrate metabolic process | 44882.2 | 6.63 | 64 | 4 |
| 91 | O35454 | Chloride transport protein 6 | transporter activity | membrane | regulation of biological process | 96917 | 6.62 | 62 | 6 |
| 527 | P07310 | Creatine kinase M-type | catalytic activity | cytoplasm | metabolic process | 43017.8 | 6.58 | 74 | 4 |
| 602 | Q64442 | Sorbitol dehydrogenase | oxidoreductase activity | mitochondrion | carbohydrate metabolic process | 38224.7 | 6.56 | 93 | 4 |
| 146 | P48678 | Prelamin-A/C | structural molecule activity | nucleus | cell communication | 73861 | 6.54 | 62 | 7 |
| 144 | P48678 | Prelamin-A/C | structural molecule activity | nucleus | cell communication | 73861 | 6.54 | 72 | 8 |
| 141 | P48678 | Prelamin-A/C | structural molecule activity | nucleus | cell communication | 73861 | 6.54 | 107 | 10 |
| 139 | P48678 | Prelamin-A/C | structural molecule activity | nucleus | cell communication | 73861 | 6.54 | 115 | 11 |
| 147 | P48678 | Prelamin-A/C | structural molecule activity | nucleus | cell communication | 73861 | 6.54 | 125 | 4 |
| 137 | P48678 | Prelamin-A/C | structural molecule activity | nucleus | cell communication | 73861 | 6.54 | 144 | 13 |
| 81 | Q811J3 | Iron-responsive element-binding protein 2 | nucleotide binding | cytoplasm | metabolic process | 104853 | 6.5 | 87 | 11 |
| 80 | Q811J3 | Iron-responsive element-binding protein 2 | nucleotide binding | cytoplasm | metabolic process | 104853 | 6.5 | 142 | 15 |
| 686 | P00920 | Carbonic anhydrase 2 | ion binding | cytoplasm | response to stimulus | 29129 | 6.49 | 88 | 6 |
| 687 | P00920 | Carbonic anhydrase 2 | ion binding | cytoplasm | response to stimulus | 29129 | 6.49 | 160 | 4 |
| 689 | P13634 | Carbonic anhydrase 1 | ion binding | cytoplasm | response to stimulus | 28883 | 6.48 | 63 | 6 |
| 190 | Q9R1E0 | Forkhead box protein O1 | protein binding | cytoplasm | regulation of biological process | 69474 | 6.47 | 110 | 5 |
| 189 | Q9R1E0 | Forkhead box protein O1 | protein binding | cytoplasm | regulation of biological process | 69474 | 6.47 | 132 | 4 |
| 220 | Q8BUG5 | Calcyphosin-2 | structural molecule activity | cytoplasm | regulation of biological process | 62686 | 6.47 | 97 | 7 |
| 431 | P47739 | Aldehyde dehydrogenase, dimeric NADP-preferring | oxidoreductase activity | mitochondrion | metabolic process | 50317 | 6.47 | 156 | 5 |
| 432 | P47739 | Aldehyde dehydrogenase, dimeric NADP-preferring | oxidoreductase activity | mitochondrion | metabolic process | 50317 | 6.47 | 156 | 5 |
| 811 | Q923D2 | Flavin reductase (NADPH) | oxidoreductase activity | cytoplasm | oxidation-reduction process | 22052 | 6.47 | 72 | 7 |
| 810 | Q923D2 | Flavin reductase (NADPH) | oxidoreductase activity | cytoplasm | oxidation-reduction process | 22052 | 6.47 | 78 | 6 |
| 807 | Q923D2 | Flavin reductase (NADPH) | oxidoreductase activity | cytoplasm | oxidation-reduction process | 22052 | 6.47 | 85 | 7 |
| 806 | Q923D2 | Flavin reductase (NADPH) | oxidoreductase activity | cytoplasm | oxidation-reduction process | 22052 | 6.47 | 98 | 9 |
| 804 | Q923D2 | Flavin reductase (NADPH) | oxidoreductase activity | cytoplasm | oxidation-reduction process | 22052 | 6.47 | 111 | 7 |
| 354 | P32921 | Tryptophanyl-tRNA synthetase, cytoplasmic | nucleotide binding | cytoplasm | multicellular organismal process | 54323 | 6.44 | 61 | 6 |
| 711 | Q03401 | Cysteine-rich secretory protein 1 | enzyme regulator activity | extracellular region | regulation of biological process | 27604.1 | 6.44 | 69 | 4 |
| 710 | Q91XA3 | Cysteine-rich secretory protein 1 | enzyme regulator activity | extracellular region | regulation of biological process | 27604.1 | 6.44 | 97 | 4 |
| 351 | Q61553 | Fascin | protein binding | cytoplasm | organelle organization | 54342 | 6.43 | 103 | 9 |
| 616 | Q8CG76 | Aflatoxin B1 aldehyde reductase member 2 | oxidoreductase activity | cytoplasm | oxidation-reduction process | 37652.6 | 6.42 | 61 | 5 |
| 612 | Q8CG76 | Aflatoxin B1 aldehyde reductase member 2 | oxidoreductase activity | cytoplasm | oxidation-reduction process | 37652.6 | 6.42 | 67 | 4 |
| 614 | Q8CG76 | Aflatoxin B1 aldehyde reductase member 2 | oxidoreductase activity | cytoplasm | oxidation-reduction process | 37652.6 | 6.42 | 93 | 5 |
| 223 | Q60864 | Stress-induced-phosphoprotein 1 | protein binding | cytoplasm | regulation of biological process | 62542.4 | 6.4 | 77 | 5 |
| 221 | Q60864 | Stress-induced-phosphoprotein 1 | protein binding | cytoplasm | regulation of biological process | 62542.4 | 6.4 | 133 | 12 |
| 451 | P17182 | Alpha-enolase | ion binding | cytoplasm | carbohydrate metabolic process | 47453 | 6.37 | 72 | 4 |
| 452 | P17182 | Alpha-enolase | ion binding | cytoplasm | carbohydrate metabolic process | 47453 | 6.37 | 73 | 8 |
| 448 | P17182 | Alpha-enolase | ion binding | cytoplasm | carbohydrate metabolic process | 47453 | 6.37 | 134 | 5 |
| 447 | P17182 | Alpha-enolase | ion binding | cytoplasm | carbohydrate metabolic process | 47453 | 6.37 | 149 | 6 |
| 455 | P17182 | Alpha-enolase | ion binding | cytoplasm | carbohydrate metabolic process | 47453 | 6.37 | 153 | 4 |
| 454 | P17182 | Alpha-enolase | ion binding | cytoplasm | carbohydrate metabolic process | 47453 | 6.37 | 157 | 4 |
| 954 | P70349 | Histidine triad nucleotide-binding protein 1 | catalytic activity | cytoplasm | nucleotide metabolic process | 13768.1 | 6.36 | 61 | 4 |
| 953 | P70349 | Histidine triad nucleotide-binding protein 1 | catalytic activity | cytoplasm | nucleotide metabolic process | 13768.1 | 6.36 | 64 | 5 |
| 951 | P70349 | Histidine triad nucleotide-binding protein 1 | catalytic activity | cytoplasm | nucleotide metabolic process | 13768.1 | 6.36 | 99 | 6 |
| 950 | P70349 | Histidine triad nucleotide-binding protein 1 | catalytic activity | cytoplasm | nucleotide metabolic process | 13768.1 | 6.36 | 112 | 5 |
| 733 | Q80W21 | Glutathione S-transferase Mu 7 | transferase activity | cytoplasm | metabolic process | 25692.9 | 6.34 | 87 | 4 |
| 862 | Q99LX0 | Protein deglycase DJ | antioxidant activity | cytoplasm | regulation of biological process | 20008.4 | 6.32 | 61 | 5 |
| 861 | Q99LX0 | Protein deglycase DJ | antioxidant activity | cytoplasm | regulation of biological process | 20008.4 | 6.32 | 68 | 5 |
| 860 | Q99LX0 | Protein deglycase DJ | antioxidant activity | cytoplasm | regulation of biological process | 20008.4 | 6.32 | 83 | 5 |
| 859 | Q99LX0 | Protein deglycase DJ | antioxidant activity | cytoplasm | regulation of biological process | 20008.4 | 6.32 | 88 | 9 |
| 47 | P01027 | Complement C3 precursor | enzyme regulator activity | vesicle | regulation of biological process | 184063 | 6.3 | 134 | 15 |
| 219 | Q9CWJ9 | Bifunctional purine biosynthesis protein PURH | protein binding | cytoplasm | regulation of biological process | 64177 | 6.3 | 110 | 9 |
| 218 | Q9CWJ9 | Bifunctional purine biosynthesis protein PURH | protein binding | cytoplasm | regulation of biological process | 64177 | 6.3 | 118 | 11 |
| 869 | P08030 | Adenine phosphoribosyltransferase | nucleotide binding | cytoplasm | response to stimulus | 19580 | 6.3 | 62 | 4 |
| 618 | Q8K157 | Aldose 1-epimerase | oxidoreductase activity | cytoplasm | metabolic process | 37644 | 6.28 | 109 | 8 |
| 617 | Q8K157 | Aldose 1-epimerase | oxidoreductase activity | cytoplasm | metabolic process | 37644 | 6.28 | 144 | 10 |
| 603 | Q8R059 | UDP-glucose 4-epimerase | catalytic activity | vesicle | carbohydrate metabolic process | 38200 | 6.27 | 62 | 5 |
| 136 | Q8BY35 | FYVE, RhoGEF and PH domain-containing protein 2 | ion binding | cytoplasm | cell communication | 74586 | 6.26 | 61 | 5 |
| 59 | Q8K1H1 | Tudor domain-containing protein 7 | nucleotide binding | cytoplasm | regulation of biological process | 122096 | 6.25 | 60 | 7 |
| 54 | Q8K1H1 | Tudor domain-containing protein 7 | nucleotide binding | cytoplasm | regulation of biological process | 122096 | 6.25 | 63 | 9 |
| 199 | P26041 | Moesin | structural molecule activity | cytoplasm | cell adhesion | 67593 | 6.24 | 67 | 7 |
| 667 | Q91X91 | Nicotinate-nucleotide pyrophosphorylase | catalytic activity | cytoplasm | nucleotide metabolic process | 31510 | 6.24 | 61 | 5 |
| 666 | Q91X91 | Nicotinate-nucleotide pyrophosphorylase | catalytic activity | cytoplasm | nucleotide metabolic process | 31510 | 6.24 | 62 | 5 |
| 665 | Q91X91 | Nicotinate-nucleotide pyrophosphorylase | catalytic activity | cytoplasm | nucleotide metabolic process | 31510 | 6.24 | 65 | 5 |
| 932 | P08228 | Superoxide dismutase [Cu-Zn] | antioxidant activity | cytoplasm | metabolic process | 15954.8 | 6.23 | 68 | 4 |
| 931 | P08228 | Superoxide dismutase [Cu-Zn] | antioxidant activity | cytoplasm | metabolic process | 15954.8 | 6.23 | 77 | 4 |
| 927 | P08228 | Superoxide dismutase [Cu‐Zn] | antioxidant activity | cytoplasm | metabolic process | 15954.8 | 6.23 | 120 | 4 |
| 933 | P08228 | Superoxide dismutase [Cu-Zn] | antioxidant activity | cytoplasm | metabolic process | 15954.8 | 6.23 | 120 | 4 |
| 929 | P08228 | Superoxide dismutase [Cu-Zn] | antioxidant activity | cytoplasm | metabolic process | 15954.8 | 6.23 | 154 | 5 |
| 930 | P08228 | Superoxide dismutase [Cu-Zn] | antioxidant activity | cytoplasm | metabolic process | 15954.8 | 6.23 | 154 | 4 |
| 539 | P61164 | Alpha-centractin | structural molecule activity | cytoplasm | regulation of biological process | 42586 | 6.19 | 60 | 4 |
| 776 | Q8BXL7 | ADP-ribosylation factor-related protein 1 | hydrolase activity | membrane | metabolic process | 22644 | 6.17 | 61 | 4 |
| 117 | Q1HFZ0 | tRNA (cytosine(34)-C(5))-methyltransferase | transferase activity | cytoplasm | metabolic process | 85397 | 6.16 | 66 | 6 |
| 630 | P14152 | Malate dehydrogenase, cytoplasmic | catalytic activity | cytoplasm | carbohydrate metabolic process | 36488.1 | 6.1599 | 154 | 4 |
| 629 | P14152 | Malate dehydrogenase, cytoplasmic | catalytic activity | cytoplasm | carbohydrate metabolic process | 36488.1 | 6.1599 | 180 | 4 |
| 82 | Q9WVL6 | Exostosin-like 3 | transferase activity | membrane | protein metabolic process | 104407 | 6.15 | 62 | 5 |
| 329 | Q9CZS1 | Aldehyde dehydrogenase X | oxidoreductase activity | mitochondrion | metabolic process | 55366 | 6.13 | 94 | 8 |
| 205 | O54775 | WNT1-inducible-signaling pathway protein 1 | protein binding | cytoplasm | cell adhesion | 66233 | 6.12 | 62 | 4 |
| 210 | O88342 | WD repeat protein 1 | protein binding | cytoplasm | cell adhesion | 66233 | 6.12 | 81 | 8 |
| 209 | O88342 | WD repeat protein 1 | protein binding | cytoplasm | cell adhesion | 66233 | 6.12 | 87 | 9 |
| 208 | O88342 | WD repeat protein 1 | protein binding | cytoplasm | cell adhesion | 66233 | 6.12 | 93 | 8 |
| 206 | O88342 | WD repeat protein 1 | protein binding | cytoplasm | cell adhesion | 66233 | 6.12 | 100 | 10 |
| 282 | Q61753 | D-3-phosphoglycerate dehydrogenase | oxidoreductase activity | cytoplasm | metabolic process | 56549.1 | 6.12 | 70 | 5 |
| 281 | Q61753 | D-3-phosphoglycerate dehydrogenase | oxidoreductase activity | cytoplasm | metabolic process | 56549.1 | 6.12 | 95 | 8 |
| 772 | P14602 | Heat shock protein beta-1 | protein binding | cytoplasm | response to stimulus | 22999 | 6.12 | 84 | 6 |
| 771 | P14602 | Heat shock protein beta-1 | protein binding | cytoplasm | response to stimulus | 22999 | 6.12 | 85 | 6 |
| 770 | P14602 | Heat shock protein beta-1 | protein binding | cytoplasm | response to stimulus | 22999 | 6.12 | 94 | 7 |
| 765 | P14602 | Heat shock protein beta-1 | protein binding | cytoplasm | response to stimulus | 22999 | 6.12 | 109 | 4 |
| 766 | P14602 | Heat shock protein beta-1 | protein binding | cytoplasm | response to stimulus | 22999 | 6.12 | 109 | 5 |
| 769 | P14602 | Heat shock protein beta-1 | protein binding | cytoplasm | response to stimulus | 22999 | 6.12 | 114 | 7 |
| 768 | P14602 | Heat shock protein beta-1 | protein binding | cytoplasm | response to stimulus | 22999 | 6.12 | 116 | 6 |
| 767 | P14602 | Heat shock protein beta-1 | protein binding | cytoplasm | response to stimulus | 22999 | 6.12 | 139 | 8 |
| 773 | P14602 | Heat shock protein beta-1 | protein binding | cytoplasm | response to stimulus | 22999 | 6.12 | 149 | 5 |
| 774 | P14602 | Heat shock protein beta-1 | protein binding | cytoplasm | response to stimulus | 22999 | 6.12 | 149 | 5 |
| 446 | P50247 | Adenosylhomocysteinase | hydrolase activity | cytoplasm | metabolic process | 47526 | 6.08 | 118 | 9 |
| 120 | Q711T7 | Glutamine-dependent NAD(+) synthetase | catalytic activity | cytoplasm | regulation of biological process | 81652 | 6.07 | 92 | 5 |
| 363 | Q6DTY7 | 6-phosphofructo-2-kinase/fructose-2,6-bisphosphatase 4 | nucleotide binding | cytoplasm | carbohydrate metabolic process | 54066 | 6.07 | 97 | 9 |
| 327 | Q9DBF1 | Alpha-aminoadipic semialdehyde dehydrogenase | oxidoreductase activity | cytoplasm | metabolic process | 55817 | 6.06 | 103 | 9 |
| 94 | Q9CQ33 | Leucine-zipper-like transcriptional regulator 1 | nucleotide binding | nucleus | regulation of biological process | 94283 | 6.04 | 60 | 5 |
| 107 | Q61624 | Zinc finger protein 148 | nucleotide binding | nucleus | multicellular organismal process | 88695 | 6.03 | 69 | 6 |
| 148 | Q8BYW1 | Rho GTPase-activating protein 25 | enzyme regulator activity | cytoplasm | cell communication | 73337 | 6.03 | 60 | 8 |
| 77 | Q02788 | Collagen alpha-2(VI) chain | structural molecule activity | extracellular matrix | cell adhesion | 110265.7 | 6.01 | 121 | 5 |
| 78 | Q02788 | Collagen alpha-2(VI) chain | structural molecule activity | extracellular matrix | cell adhesion | 110265.7 | 6.01 | 134 | 4 |
| 681 | Q9R1P4 | Proteasome subunit alpha type-1 | hydrolase activity | proteasome | protein metabolic process | 29527 | 6 | 163 | 4 |
| 253 | Q542X7 | Chaperonin subunit 2 (Beta), isoform CRA_a | protein binding | cytoplasm | protein metabolic process | 57441 | 5.97 | 87 | 4 |
| 257 | P80314 | T-complex protein 1 subunit beta | nucleotide binding | cytoplasm | protein metabolic process | 57411.1 | 5.97 | 89 | 5 |
| 256 | P80314 | T-complex protein 1 subunit beta | nucleotide binding | cytoplasm | protein metabolic process | 57411.1 | 5.97 | 124 | 10 |
| 254 | P80314 | T-complex protein 1 subunit beta | nucleotide binding | cytoplasm | protein metabolic process | 57411.1 | 5.97 | 135 | 4 |
| 258 | P80314 | T-complex protein 1 subunit beta | nucleotide binding | cytoplasm | protein metabolic process | 57411.1 | 5.97 | 150 | 5 |
| 663 | P53810 | Phosphatidylinositol transfer protein alpha isoform | binding | cytoplasm | transport | 31742 | 5.97 | 65 | 5 |
| 791 | Q8R527 | Rho-related GTP-binding protein RhoQ | protein binding | cytoplasm | metabolic process | 22303 | 5.94 | 60 | 4 |
| 232 | P63038 | 60 kDa heat shock protein, mitochondrial | antioxidant activity | cytoplasm | regulation of biological process | 61088 | 5.91 | 70 | 4 |
| 231 | P63038 | 60 kDa heat shock protein, mitochondrial | antioxidant activity | cytoplasm | regulation of biological process | 61088 | 5.91 | 72 | 4 |
| 230 | P63038 | 60 kDa heat shock protein, mitochondrial | antioxidant activity | cytoplasm | regulation of biological process | 61088 | 5.91 | 144 | 4 |
| 233 | P63038 | 60 kDa heat shock protein, mitochondrial | antioxidant activity | cytoplasm | regulation of biological process | 61088 | 5.91 | 163 | 4 |
| 868 | Q9CZ69 | CKLF-like MARVEL transmembrane domain-containing protein 6 | protein binding | membrane | response to stimulus | 19824 | 5.91 | 71 | 5 |
| 173 | P16627 | Heat shock 70 kDa protein 1-like | protein binding | cytoplasm | response to stimulus | 70593 | 5.9 | 62 | 4 |
| 435 | P70333 | Heterogeneous nuclear ribonucleoprotein H2 | nucleotide binding | nucleus | regulation of biological process | 49248 | 5.89 | 120 | 11 |
| 475 | Q99JW2 | Aminoacylase-1 | hydrolase activity | cytoplasm | metabolic process | 45980 | 5.89 | 126 | 9 |
| 474 | Q99JW2 | Aminoacylase-1 | hydrolase activity | cytoplasm | metabolic process | 45980 | 5.89 | 146 | 13 |
| 473 | Q99JW2 | Aminoacylase-1 | hydrolase activity | cytoplasm | metabolic process | 45980 | 5.89 | 148 | 10 |
| 471 | Q99JW2 | Aminoacylase-1 | hydrolase activity | cytoplasm | metabolic process | 45980 | 5.89 | 150 | 5 |
| 739 | Q60631 | Growth factor receptor-bound protein 2 | protein binding | cytoplasm | organelle organization | 25222 | 5.89 | 71 | 7 |
| 272 | P27773 | Protein disulfide-isomerase A3 | oxidoreductase activity | cytoplasm | regulation of biological process | 56642.7 | 5.88 | 70 | 8 |
| 273 | P27773 | Protein disulfide-isomerase A3 | oxidoreductase activity | cytoplasm | regulation of biological process | 56642.7 | 5.88 | 70 | 6 |
| 271 | P27773 | Protein disulfide-isomerase A3 | oxidoreductase activity | cytoplasm | regulation of biological process | 56642.7 | 5.88 | 84 | 5 |
| 270 | P27773 | Protein disulfide-isomerase A3 | oxidoreductase activity | cytoplasm | regulation of biological process | 56642.7 | 5.88 | 96 | 9 |
| 265 | P27773 | Protein disulfide-isomerase A3 | oxidoreductase activity | cytoplasm | regulation of biological process | 56642.7 | 5.88 | 105 | 5 |
| 266 | P27773 | Protein disulfide-isomerase A3 | oxidoreductase activity | cytoplasm | regulation of biological process | 56642.7 | 5.88 | 105 | 5 |
| 267 | P27773 | Protein disulfide-isomerase A3 | oxidoreductase activity | cytoplasm | regulation of biological process | 56642.7 | 5.88 | 105 | 4 |
| 278 | P27773 | Protein disulfide-isomerase A3 | oxidoreductase activity | cytoplasm | regulation of biological process | 56642.7 | 5.88 | 125 | 4 |
| 279 | P27773 | Protein disulfide-isomerase A3 | oxidoreductase activity | cytoplasm | regulation of biological process | 56642.7 | 5.88 | 125 | 4 |
| 274 | P27773 | Protein disulfide-isomerase A3 | oxidoreductase activity | cytoplasm | regulation of biological process | 56642.7 | 5.88 | 139 | 5 |
| 277 | P27773 | Protein disulfide-isomerase A3 | oxidoreductase activity | cytoplasm | regulation of biological process | 56642.7 | 5.88 | 154 | 5 |
| 276 | P27773 | Protein disulfide-isomerase A3 | oxidoreductase activity | cytoplasm | regulation of biological process | 56642.7 | 5.88 | 155 | 5 |
| 275 | P27773 | Protein disulfide-isomerase A3 | oxidoreductase activity | cytoplasm | regulation of biological process | 56642.7 | 5.88 | 160 | 4 |
| 268 | P27773 | Protein disulfide-isomerase A3 | oxidoreductase activity | cytoplasm | regulation of biological process | 56642.7 | 5.88 | 191 | 16 |
| 409 | P17563 | Selenium-binding protein 1 | binding | cytoplasm | transport | 52349 | 5.88 | 64 | 6 |
| 406 | P17563 | Selenium-binding protein 1 | binding | cytoplasm | transport | 52349 | 5.88 | 89 | 9 |
| 402 | P17563 | Selenium-binding protein 1 | binding | cytoplasm | transport | 52349 | 5.88 | 103 | 10 |
| 399 | P17563 | Selenium-binding protein 1 | binding | cytoplasm | transport | 52349 | 5.88 | 108 | 10 |
| 650 | Q8K183 | Pyridoxal kinase | transferase activity | cytoplasm | metabolic process | 34992 | 5.88 | 84 | 6 |
| 195 | O35227 | Disintegrin and metalloproteinase domain-containing protein 7 | structural molecule activity | membrane | protein metabolic process | 68671 | 5.86 | 155 | 11 |
| 331 | Q61247 | Alpha-2-antiplasmin | structural molecule activity | extracellular matrix | cell adhesion | 54971 | 5.86 | 93 | 6 |
| 65 | Q2PFD7 | PH and SEC7 domain-containing protein 3 | protein binding | membrane | cell communication | 114651 | 5.85 | 64 | 5 |
| 738 | P70195 | Proteasome subunit beta type 7 | hydrolase activity | proteasome | protein metabolic process | 25235 | 5.84 | 61 | 6 |
| 191 | P26040 | Ezrin | protein binding | cytoplasm | regulation of biological process | 69363.6 | 5.83 | 93 | 4 |
| 192 | P26040 | Ezrin | protein binding | cytoplasm | regulation of biological process | 69302.6 | 5.83 | 90 | 4 |
| 413 | P80317 | T-complex protein 1 subunit zeta | nucleotide binding | cytoplasm | protein metabolic process | 52004 | 5.83 | 65 | 6 |
| 648 | Q91VM9 | Inorganic pyrophosphatase 2 | ion binding | cytoplasm | metabolic process | 35122 | 5.83 | 124 | 9 |
| 813 | O89061 | Delta-aminolevulinic acid dehydratase | catalytic activity | cytoplasm | metabolic process | 21905 | 5.83 | 72 | 4 |
| 778 | Q8VCR7 | Abhydrolase domain-containing protein 14B | hydrolase activity | cytoplasm | metabolic process | 22436 | 5.82 | 65 | 5 |
| 779 | Q8VCR7 | Abhydrolase domain-containing protein 13B | hydrolase activity | cytoplasm | metabolic process | 22436 | 5.82 | 110 | 5 |
| 62 | Q64727 | Vinculin | structural molecule activity | cytoplasm | cell adhesion | 116513 | 5.77 | 105 | 12 |
| 63 | Q64727 | Vinculin | structural molecule activity | cytoplasm | cell adhesion | 116513 | 5.77 | 137 | 4 |
| 64 | Q64727 | Vinculin | structural molecule activity | cytoplasm | cell adhesion | 116513 | 5.77 | 137 | 4 |
| 61 | Q64727 | Vinculin | structural molecule activity | cytoplasm | cell adhesion | 116513 | 5.77 | 166 | 4 |
| 956 | P07309 | Transthyretin | protein binding | vesicle | transport | 13631 | 5.77 | 104 | 5 |
| 193 | Q546G4 | Albumin 1 | protein binding | cytoplasm | regulation of biological process | 68693.7 | 5.75 | 94 | 5 |
| 382 | Q9CZ13 | Cytochrome b-c1 complex subunit 2, mitochondrial | transporter activity | mitochondrion | transport | 53420 | 5.75 | 70 | 4 |
| 390 | Q9CZ13 | Cytochrome b-c1 complex subunit 1, mitochondrial | transporter activity | mitochondrion | transport | 53420 | 5.75 | 148 | 5 |
| 697 | P97371 | Proteasome activator complex subunit 1 | hydrolase activity | proteasome | protein metabolic process | 28654 | 5.73 | 61 | 6 |
| 696 | P97371 | Proteasome activator complex subunit 1 | hydrolase activity | proteasome | protein metabolic process | 28654 | 5.73 | 62 | 5 |
| 695 | P97371 | Proteasome activator complex subunit 1 | hydrolase activity | proteasome | protein metabolic process | 28654 | 5.73 | 144 | 12 |
| 239 | P80316 | T-complex protein 1 subunit epsilon | nucleotide binding | cytoplasm | protein metabolic process | 59454 | 5.72 | 81 | 8 |
| 730 | P57759 | Endoplasmic reticulum resident protein 29 | protein binding | vesicle | response to stimulus | 25721 | 5.71 | 126 | 4 |
| 731 | P57759 | Endoplasmic reticulum resident protein 29 | protein binding | vesicle | response to stimulus | 25721 | 5.71 | 126 | 4 |
| 744 | O08709 | Peroxiredoxin-6 | antioxidant activity | cytoplasm | metabolic process | 24885 | 5.71 | 74 | 6 |
| 743 | O08709 | Peroxiredoxin-6 | antioxidant activity | cytoplasm | metabolic process | 24885 | 5.71 | 111 | 7 |
| 803 | Q61686 | Chromobox protein homolog 5 | protein binding | nucleus | regulation of biological process | 22171 | 5.71 | 61 | 5 |
| 636 | P16125 | L-lactate dehydrogenase B chain | protein binding | cytoplasm | carbohydrate metabolic process | 36417 | 5.7 | 72 | 6 |
| 635 | P16125 | L-lactate dehydrogenase B chain | protein binding | cytoplasm | carbohydrate metabolic process | 36417 | 5.7 | 121 | 8 |
| 688 | Q9WU84 | Copper chaperone for superoxide dismutase | antioxidant activity | cytoplasm | metabolic process | 28911 | 5.7 | 77 | 4 |
| 29 | Q8BTM8 | Filamin-A | structural molecule activity | cytoplasm | organelle organization | 280914 | 5.68 | 60 | 9 |
| 31 | Q8BTM8 | Filamin-A | structural molecule activity | cytoplasm | organelle organization | 280914 | 5.68 | 95 | 4 |
| 30 | Q8BTM8 | Filamin-A | structural molecule activity | cytoplasm | organelle organization | 280914 | 5.68 | 96 | 4 |
| 217 | Q99MX0 | Transketolase-like protein 1 | catalytic activity | cytoplasm | metabolic process | 65202 | 5.67 | 141 | 4 |
| 833 | Q9R0Y5 | Adenylate kinase isoenzyme 1 | nucleotide binding | cytoplasm | nucleotide metabolic process | 21526 | 5.67 | 94 | 4 |
| 836 | Q9R0Y5 | Adenylate kinase isoenzyme 1 | nucleotide binding | cytoplasm | nucleotide metabolic process | 21526 | 5.67 | 102 | 8 |
| 834 | Q9R0Y5 | Adenylate kinase isoenzyme 1 | nucleotide binding | cytoplasm | nucleotide metabolic process | 21526 | 5.67 | 110 | 7 |
| 670 | Q9CWS0 | N(G),N(G)-dimethylarginine dimethylaminohydrolase 2 | hydrolase activity | cytoplasm | regulation of biological process | 31229 | 5.64 | 105 | 8 |
| 672 | Q9CWS0 | N(G),N(G)-dimethylarginine dimethylaminohydrolase 2 | hydrolase activity | cytoplasm | regulation of biological process | 31229 | 5.64 | 109 | 5 |
| 671 | Q9CWS0 | N(G),N(G)-dimethylarginine dimethylaminohydrolase 2 | hydrolase activity | cytoplasm | regulation of biological process | 31229 | 5.64 | 110 | 5 |
| 914 | Q5EBG6 | Heat-shock protein beta-6 | protein binding | cytoplasm | response to stimulus | 17510 | 5.64 | 110 | 6 |
| 60 | Q80Z25 | Oral-facial-digital syndrome 1 protein | protein binding | cytoplasm | organelle organization | 117272 | 5.63 | 62 | 7 |
| 610 | P18242 | Cathepsin D | hydrolase activity | vesicle | cell communication | 37983 | 5.63 | 60 | 5 |
| 609 | P18242 | Cathepsin D | hydrolase activity | vesicle | cell communication | 37983 | 5.63 | 62 | 5 |
| 816 | Q9D892 | Inosine triphosphate pyrophosphatase | hydrolase activity | cytoplasm | nucleotide metabolic process | 21751 | 5.6 | 85 | 5 |
| 280 | Q62148 | Retinal dehydrogenase 2 | oxidoreductase activity | cytoplasm | metabolic process | 56625 | 5.58 | 89 | 6 |
| 411 | P51855 | Glutathione synthetase | catalytic activity | cytoplasm | metabolic process | 52082 | 5.57 | 114 | 9 |
| 412 | P51855 | Glutathione synthetase | catalytic activity | cytoplasm | metabolic process | 52082 | 5.57 | 114 | 9 |
| 677 | P67778 | Prohibitin | protein binding | cytoplasm | multicellular organismal process | 29801 | 5.57 | 93 | 7 |
| 574 | P63260 | Actin, cytoplasmic 2 | structural molecule activity | cytoskeleton | organelle organization | 40992.5 | 5.56 | 75 | 4 |
| 721 | Q9CQ60 | 6‐phosphogluconolactonase | hydrolase activity | cytoplasm | carbohydrate metabolic process | 27106 | 5.56 | 157 | 5 |
| 659 | Q9Z0S1 | 3'(2'),5'-bisphosphate nucleotidase 1 | ion binding | nucleus | nucleotide metabolic process | 33044 | 5.54 | 60 | 4 |
| 83 | Q8BHN3 | Neutral alpha-glucosidase AB | catalytic activity | vesicle | metabolic process | 103476 | 5.52 | 89 | 10 |
| 879 | Q9DCX2 | ATP synthase subunit d, mitochondrial | hydrolase activity | mitochondrion | metabolic process | 18795 | 5.52 | 96 | 4 |
| 881 | Q9DCX2 | ATP synthase subunit d, mitochondrial | hydrolase activity | mitochondrion | metabolic process | 18795 | 5.52 | 119 | 4 |
| 576 | P59913 | Protein-L-isoaspartate O-methyltransferase domain-containing protein 1 | transferase activity | cytoplasm | metabolic process | 40536 | 5.47 | 95 | 8 |
| 674 | P47757 | F-actin capping protein subunit beta | structural molecule activity | membrane | multicellular organismal process | 31194 | 5.47 | 125 | 11 |
| 86 | Q80ZX8 | Sperm-associated antigen 1 | nucleotide binding | cytoplasm | metabolic process | 100607 | 5.46 | 100 | 8 |
| 421 | Q06890 | Clusterin | protein binding | cytoplasm | regulation of biological process | 51622.6 | 5.46 | 89 | 4 |
| 188 | Q8BHI4 | Kelch repeat and BTB domain-containing protein 3 | nucleotide binding | cytoplasm | response to stimulus | 69505 | 5.44 | 61 | 5 |
| 197 | P38647 | Stress-70 protein, mitochondrial | protein binding | cytoplasm | transport | 68503 | 5.44 | 76 | 7 |
| 196 | P38647 | Stress-70 protein, mitochondrial | protein binding | cytoplasm | transport | 68503 | 5.44 | 79 | 4 |
| 198 | P38647 | Stress-70 protein, mitochondrial | protein binding | cytoplasm | transport | 68503 | 5.44 | 163 | 4 |
| 481 | P07758 | Alpha-1-antitrypsin 1-1 | enzyme regulator activity | extracellular region | metabolic process | 45973.5 | 5.44 | 124 | 10 |
| 977 | Q5XG73 | Acyl-CoA-binding domain-containing protein 7 | ion binding | cytoplasm | regulation of biological process | 9921 | 5.44 | 93 | 6 |
| 395 | Q9D1A2 | Cytosolic non-specific dipeptidase | hydrolase activity | cytoplasm | metabolic process | 52602 | 5.43 | 65 | 4 |
| 394 | Q9D1A2 | Cytosolic non-specific dipeptidase | hydrolase activity | cytoplasm | metabolic process | 52602 | 5.43 | 126 | 11 |
| 643 | P97429 | Annexin A4 | structural molecule activity | vesicle | response to stimulus | 35762 | 5.42 | 135 | 9 |
| 521 | Q9JK98 | Clusterin | protein binding | cytoplasm | regulation of biological process | 43081.9 | 5.41 | 93 | 4 |
| 700 | O88456 | Calpain small subunit 1 | protein binding | cytoplasm | multicellular organismal process | 28444 | 5.41 | 61 | 4 |
| 540 | Q04447 | Creatine kinase B-type | catalytic activity | cytoplasm | metabolic process | 42555 | 5.4 | 81 | 5 |
| 691 | Q00623 | Apolipoprotein A-I precursor | protein binding | extracellular region | transport | 28797 | 5.4 | 90 | 7 |
| 690 | Q00623 | Apolipoprotein A-I precursor | protein binding | extracellular region | transport | 28797 | 5.4 | 120 | 10 |
| 44 | O08638 | Myosin-11 | structural molecule activity | cytoskeleton | organelle organization | 226887 | 5.37 | 119 | 4 |
| 45 | O08638 | Myosin-11 | structural molecule activity | cytoskeleton | organelle organization | 226887 | 5.37 | 119 | 4 |
| 52 | Q6ZQ73 | Cullin-associated NEDD8-dissociated protein 2 | protein binding | nucleus | regulation of biological process | 135415 | 5.37 | 60 | 7 |
| 169 | P63017 | Heat shock cognate 71 kDa protein | protein binding | cytoplasm | response to stimulus | 70793.2 | 5.37 | 64 | 5 |
| 168 | P63017 | Heat shock cognate 71 kDa protein | protein binding | cytoplasm | response to stimulus | 70793.2 | 5.37 | 66 | 5 |
| 171 | P63017 | Heat shock cognate 71 kDa protein | protein binding | cytoplasm | response to stimulus | 70793.2 | 5.37 | 76 | 4 |
| 170 | P63017 | Heat shock cognate 71 kDa protein | protein binding | cytoplasm | response to stimulus | 70793.2 | 5.37 | 79 | 8 |
| 172 | P63017 | Heat shock cognate 71 kDa protein | protein binding | cytoplasm | response to stimulus | 70793.2 | 5.37 | 160 | 5 |
| 365 | Q8K2J9 | BTB/POZ domain-containing protein 6 | protein binding | cytoplasm | regulation of biological process | 53808 | 5.37 | 61 | 5 |
| 202 | P21619 | Lamin-B2 | structural molecule activity | nucleus | regulation of biological process | 66877 | 5.36 | 60 | 7 |
| 538 | Q61852 | Smooth muscle gamma-actin | structural molecule activity | cytoskeleton | regulation of biological process | 42852.3 | 5.36 | 79 | 4 |
| 661 | P47753 | F-actin-capping protein subunit alpha-1 | structural molecule activity | membrane | multicellular organismal process | 32788 | 5.34 | 78 | 7 |
| 786 | P61089 | Ubiquitin-conjugating enzyme E2 N | protein binding | cytoplasm | protein metabolic process | 22407.6 | 5.33 | 72 | 5 |
| 790 | P61087 | Ubiquitin-conjugating enzyme E2 K | protein binding | cytoplasm | protein metabolic process | 22407.6 | 5.33 | 90 | 6 |
| 788 | P61087 | Ubiquitin-conjugating enzyme E2 K | protein binding | cytoplasm | protein metabolic process | 22407.6 | 5.33 | 98 | 5 |
| 49 | Q9Z0W3 | Nuclear pore complex protein Nup160 | nucleotide binding | nucleus | nucleotide metabolic process | 158130 | 5.32 | 63 | 6 |
| 572 | P60710 | Actin, cytoplasmic 1 | structural molecule activity | cytoskeleton | organelle organization | 41737.7 | 5.29 | 127 | 6 |
| 702 | O70435 | Proteasome subunit beta type 3 | hydrolase activity | proteasome | protein metabolic process | 28256 | 5.29 | 74 | 7 |
| 814 | P07361 | Alpha-1-acid glycoprotein 2 | protein binding | extracellular region | regulation of biological process | 21853 | 5.28 | 120 | 4 |
| 937 | P16045 | Galectin-1 | protein binding | membrane | cell adhesion | 14725 | 5.28 | 84 | 5 |
| 936 | P16045 | Galectin-1 | protein binding | membrane | cell adhesion | 14725 | 5.28 | 135 | 5 |
| 971 | P50543 | Protein S100-A11 | ion binding | cytoplasm | regulation of biological process | 11075 | 5.28 | 60 | 5 |
| 67 | Q8VDN2 | Potassium-transporting ATPase alpha chain 1 | hydrolase activity | vesicle | metabolic process | 112437 | 5.27 | 64 | 7 |
| 552 | O08797 | Protein Serpinb9 | protein binding | cytoplasm | regulation of biological process | 42232 | 5.27 | 110 | 4 |
| 608 | Q8VDQ1 | Prostaglandin reductase 2 | oxidoreductase activity | cytoplasm | oxidation-reduction process | 37991 | 5.27 | 151 | 4 |
| 891 | P60154 | Ribonuclease-like protein 9 | nucleotide binding | extracellular region | regulation of biological process | 18487 | 5.26 | 86 | 5 |
| 964 | P48428 | Tubulin-specific chaperone A | structural molecule activity | cytoskeleton | organelle organization | 12618 | 5.25 | 62 | 6 |
| 128 | Q91VD9 | NADH-ubiquinone oxidoreductase 75 kDa | oxidoreductase activity | mitochondrion | metabolic process | 77161 | 5.24 | 74 | 7 |
| 470 | Q00897 | Alpha-1-antitrypsin 1-4 | enzyme regulator activity | extracellular region | metabolic process | 45998 | 5.24 | 88 | 4 |
| 565 | P68134 | Actin, alpha skeletal muscle | structural molecule activity | cytoskeleton | organelle organization | 42051.9 | 5.23 | 77 | 4 |
| 713 | P10605 | Cathepsin B | hydrolase activity | vesicle | cell communication | 27559 | 5.23 | 61 | 6 |
| 38 | Q8VHY0 | Chondroitin sulfate proteoglycan 4 | protein binding | vesicle | multicellular organismal process | 249307 | 5.21 | 94 | 5 |
| 39 | Q8VHY0 | Chondroitin sulfate proteoglycan 4 | protein binding | vesicle | multicellular organismal process | 249307 | 5.21 | 139 | 4 |
| 379 | P31001 | Desmin | protein binding | vesicle | multicellular organismal process | 53465 | 5.21 | 83 | 8 |
| 372 | P31001 | Desmin | protein binding | vesicle | multicellular organismal process | 53465 | 5.21 | 109 | 9 |
| 420 | P21614 | Vitamin D-binding protein | binding | vesicle | transport | 51831 | 5.21 | 76 | 4 |
| 79 | Q04857 | Collagen alpha-1(VI) chain | structural molecule activity | extracellular matrix | cell adhesion | 108421.9 | 5.2 | 77 | 5 |
| 176 | Q61233 | Plastin-2 | structural molecule activity | cytoplasm | cell communication | 69973 | 5.2 | 114 | 10 |
| 175 | Q61233 | Plastin-2 | structural molecule activity | cytoplasm | cell communication | 69973 | 5.2 | 133 | 12 |
| 489 | Q91XA2 | Golgi phosphoprotein 1 | nucleotide binding | vesicle | regulation of biological process | 44299 | 5.2 | 61 | 5 |
| 829 | Q61171 | Peroxiredoxin-2 | antioxidant activity | cytoplasm | metabolic process | 21634 | 5.2 | 62 | 4 |
| 821 | Q61171 | peroxiredoxin‐2 | antioxidant activity | cytoplasm | metabolic process | 21634 | 5.2 | 98 | 5 |
| 822 | Q61171 | Peroxiredoxin-2 | antioxidant activity | cytoplasm | metabolic process | 21634 | 5.2 | 98 | 5 |
| 824 | Q61171 | Peroxiredoxin-2 | antioxidant activity | cytoplasm | metabolic process | 21634 | 5.2 | 99 | 6 |
| 823 | Q61171 | Peroxiredoxin-2 | antioxidant activity | cytoplasm | metabolic process | 21634 | 5.2 | 101 | 6 |
| 832 | Q61171 | peroxiredoxin 2 | antioxidant activity | cytoplasm | metabolic process | 21634 | 5.2 | 185 | 5 |
| 531 | Q91W90 | Thioredoxin domain-containing protein 5 | isomerase activity | vesicle | response to stimulus | 43002 | 5.19 | 70 | 7 |
| 528 | Q91W90 | Thioredoxin domain-containing protein 5 | isomerase activity | vesicle | response to stimulus | 43002 | 5.19 | 148 | 9 |
| 849 | P70296 | Phosphatidylethanolamine-binding protein 1 | enzyme regulator activity | cytoplasm | regulation of biological process | 20817.3 | 5.19 | 66 | 4 |
| 848 | P70296 | Phosphatidylethanolamine-binding protein 1 | enzyme regulator activity | cytoplasm | regulation of biological process | 20817.3 | 5.19 | 71 | 5 |
| 846 | P70296 | Phosphatidylethanolamine-binding protein 1 | enzyme regulator activity | cytoplasm | regulation of biological process | 20817.3 | 5.19 | 91 | 5 |
| 845 | P70296 | Phosphatidylethanolamine-binding protein 1 | enzyme regulator activity | cytoplasm | regulation of biological process | 20817.3 | 5.19 | 98 | 6 |
| 844 | P70296 | Phosphatidylethanolamine-binding protein 1 | enzyme regulator activity | cytoplasm | regulation of biological process | 20817.3 | 5.19 | 106 | 6 |
| 843 | P70296 | Phosphatidylethanolamine-binding protein 1 | enzyme regulator activity | cytoplasm | regulation of biological process | 20817.3 | 5.19 | 111 | 7 |
| 541 | Q9R0N0 | Galactokinase | transferase activity | cytoplasm | metabolic process | 42295 | 5.17 | 92 | 5 |
| 547 | Q9R0N0 | Galactokinase | transferase activity | cytoplasm | metabolic process | 42295 | 5.17 | 127 | 9 |
| 41 | P15508 | Spectrin beta chain, erythrocytic | protein binding | cytoplasm | organelle organization | 244967 | 5.16 | 61 | 7 |
| 167 | P08003 | Protein disulfide-isomerase A4 | oxidoreductase activity | cytoplasm | regulation of biological process | 71982 | 5.16 | 107 | 5 |
| 864 | P48026 | Diamine acetyltransferase 1 | transferase activity | cytoplasm | multicellular organismal process | 19998 | 5.16 | 102 | 5 |
| 863 | P48026 | Diamine acetyltransferase 1 | transferase activity | cytoplasm | multicellular organismal process | 19998 | 5.16 | 125 | 7 |
| 103 | Q01853 | Transitional endoplasmic reticulum ATPase | protein binding | cytoplasm | regulation of biological process | 89134 | 5.14 | 106 | 4 |
| 106 | Q01853 | Transitional endoplasmic reticulum ATPase | protein binding | cytoplasm | regulation of biological process | 89134 | 5.14 | 106 | 4 |
| 292 | P56480 | ATP synthase subunit beta, mitochondrial | hydrolase activity | mitochondrion | metabolic process | 56344.5 | 5.14 | 87 | 7 |
| 301 | P56480 | ATP synthase subunit beta, mitochondrial | hydrolase activity | mitochondrion | metabolic process | 56344.5 | 5.14 | 113 | 6 |
| 299 | P56480 | ATP synthase subunit beta, mitochondrial | hydrolase activity | mitochondrion | metabolic process | 56344.5 | 5.14 | 122 | 4 |
| 291 | P56480 | ATP synthase subunit beta, mitochondrial | hydrolase activity | mitochondrion | metabolic process | 56344.5 | 5.14 | 128 | 6 |
| 314 | Q03265 | ATP synthase subunit alpha, mitochondrial | hydrolase activity | mitochondrion | metabolic process | 56344.5 | 5.14 | 134 | 4 |
| 289 | P56480 | ATP synthase subunit beta, mitochondrial | hydrolase activity | mitochondrion | metabolic process | 56344.5 | 5.14 | 138 | 7 |
| 298 | P56480 | ATP synthase subunit beta, mitochondrial | hydrolase activity | mitochondrion | metabolic process | 56344.5 | 5.14 | 145 | 5 |
| 302 | P56480 | ATP synthase subunit beta, mitochondrial | hydrolase activity | mitochondrion | metabolic process | 56344.5 | 5.14 | 148 | 5 |
| 305 | Q03265 | ATP synthase subunit alpha, mitochondrial | hydrolase activity | mitochondrion | metabolic process | 56344.5 | 5.14 | 153 | 5 |
| 307 | Q03265 | ATP synthase subunit alpha, mitochondrial | hydrolase activity | mitochondrion | metabolic process | 56344.5 | 5.14 | 153 | 5 |
| 313 | Q03265 | ATP synthase subunit alpha, mitochondrial | hydrolase activity | mitochondrion | metabolic process | 56344.5 | 5.14 | 180 | 5 |
| 310 | Q03265 | ATP synthase subunit alpha, mitochondrial | hydrolase activity | mitochondrion | metabolic process | 56344.5 | 5.14 | 181 | 5 |
| 494 | Q99KJ8 | Dynactin subunit 2 | structural molecule activity | cytoplasm | metabolic process | 43985 | 5.14 | 102 | 4 |
| 716 | Q8K1Y4 | Kelch-like protein 15 | nucleotide binding | cytoplasm | response to stimulus | 27503 | 5.14 | 60 | 5 |
| 714 | Q8K1Y4 | Kelch-like protein 15 | nucleotide binding | cytoplasm | response to stimulus | 27503 | 5.14 | 61 | 5 |
| 715 | Q8K1Y4 | Kelch-like protein 15 | nucleotide binding | cytoplasm | response to stimulus | 27503 | 5.14 | 61 | 5 |
| 76 | Q9JKR6 | Hypoxia up-regulated protein 1 | ion binding | endoplasmic reticulum | response to stimulus | 111112.2 | 5.12 | 99 | 4 |
| 69 | Q9JKR6 | Hypoxia up-regulated protein 1 | ion binding | endoplasmic reticulum | response to stimulus | 111112.2 | 5.12 | 121 | 5 |
| 762 | Q99PT1 | Rho GDP-dissociation inhibitor 1 | enzyme regulator activity | cytoplasm | metabolic process | 23261 | 5.1 | 85 | 7 |
| 763 | Q99PT1 | Rho GDP-dissociation inhibitor 1 | enzyme regulator activity | cytoplasm | metabolic process | 23261 | 5.1 | 85 | 5 |
| 764 | Q99PT1 | Rho GDP-dissociation inhibitor 1 | enzyme regulator activity | cytoplasm | metabolic process | 23261 | 5.1 | 85 | 5 |
| 761 | Q99PT1 | Rho GDP-dissociation inhibitor 1 | enzyme regulator activity | cytoplasm | metabolic process | 23261 | 5.1 | 94 | 7 |
| 759 | Q99PT1 | Rho GDP-dissociation inhibitor 1 | enzyme regulator activity | cytoplasm | metabolic process | 23261 | 5.1 | 105 | 6 |
| 158 | P20029 | 78 kDa glucose-regulated protein | antioxidant activity | cytoplasm | regulation of biological process | 72377.5 | 5.07 | 61 | 7 |
| 161 | P20029 | 78 kDa glucose-regulated protein | antioxidant activity | cytoplasm | regulation of biological process | 72377.5 | 5.07 | 61 | 5 |
| 154 | P20029 | 78 kDa glucose-regulated protein | antioxidant activity | cytoplasm | regulation of biological process | 72377.5 | 5.07 | 65 | 5 |
| 155 | P20029 | 78 kDa glucose-regulated protein | antioxidant activity | cytoplasm | regulation of biological process | 72377.5 | 5.07 | 65 | 5 |
| 157 | P20029 | 78 kDa glucose-regulated protein | antioxidant activity | cytoplasm | regulation of biological process | 72377.5 | 5.07 | 65 | 4 |
| 160 | P20029 | 78 kDa glucose-regulated protein | antioxidant activity | cytoplasm | regulation of biological process | 72377.5 | 5.07 | 66 | 7 |
| 166 | P20029 | 78 kDa glucose-regulated protein | antioxidant activity | cytoplasm | regulation of biological process | 72377.5 | 5.07 | 86 | 6 |
| 164 | P20029 | 78 kDa glucose-regulated protein | antioxidant activity | cytoplasm | regulation of biological process | 72377.5 | 5.07 | 108 | 9 |
| 163 | P20029 | 78 kDa glucose-regulated protein | antioxidant activity | cytoplasm | regulation of biological process | 72377.5 | 5.07 | 123 | 11 |
| 948 | Q9DAK9 | 14 kDa phosphohistidine phosphatase | hydrolase activity | cytoplasm | cell communication | 13987 | 5.07 | 61 | 5 |
| 947 | Q9DAK9 | 14 kDa phosphohistidine phosphatase | hydrolase activity | cytoplasm | cell communication | 13987 | 5.07 | 77 | 6 |
| 370 | P20152 | Vimentin | structural molecule activity | cytoplasm | cell adhesion | 53524 | 5.05 | 72 | 7 |
| 368 | P20152 | Vimentin | structural molecule activity | cytoplasm | cell adhesion | 53524 | 5.05 | 120 | 11 |
| 438 | Q3THH1 | Protein disulfide-isomerase A6 | oxidoreductase activity | cytoplasm | regulation of biological process | 48626.6 | 5.05 | 123 | 4 |
| 439 | Q922R8 | Protein disulfide-isomerase A6 | oxidoreductase activity | cytoplasm | regulation of biological process | 48626.6 | 5.05 | 140 | 4 |
| 441 | Q922R8 | Protein disulfide-isomerase A6 | oxidoreductase activity | cytoplasm | regulation of biological process | 48626.6 | 5.05 | 140 | 4 |
| 912 | Q9D3D9 | ATP synthase subunit delta, mitochondrial | hydrolase activity | mitochondrion | metabolic process | 17589 | 5.03 | 173 | 4 |
| 150 | P20030 | 79 kDa glucose-regulated protein | ion binding | cytoplasm | response to stimulus | 72492 | 5.02 | 60 | 5 |
| 457 | P17183 | Gamma-enolase | ion binding | cytoplasm | carbohydrate metabolic process | 47135 | 4.99 | 62 | 5 |
| 130 | P21981 | Protein-glutamine gamma-glutamyltransferase 2 | transferase activity | cytoplasm | regulation of biological process | 76881 | 4.98 | 65 | 6 |
| 129 | P21981 | Protein-glutamine gamma-glutamyltransferase 2 | transferase activity | cytoplasm | regulation of biological process | 76881 | 4.98 | 77 | 8 |
| 433 | P05214 | Tubulin alpha-3 chain | structural molecule activity | cytoskeleton | organelle organization | 49927 | 4.98 | 102 | 8 |
| 119 | P11499 | Heat shock protein HSP 90-beta | protein binding | cytoplasm | response to stimulus | 83098 | 4.97 | 81 | 8 |
| 426 | A2AQ07 | Tubulin beta-1 chain | structural molecule activity | cytoskeleton | organelle organization | 50408 | 4.96 | 62 | 5 |
| 837 | Q9CZ19 | Myosin light chain 4 | structural molecule activity | cytoskeleton | organelle organization | 21189 | 4.96 | 95 | 5 |
| 935 | P56395 | Cytochrome b5 | transporter activity | mitochondrion | transport | 15100 | 4.94 | 68 | 4 |
| 118 | P07901 | Heat shock protein HSP 90-alpha | protein binding | cytoplasm | response to stimulus | 84603 | 4.93 | 81 | 9 |
| 693 | Q61696 | Heat shock 70 kDa protein 1A | protein binding | cytoplasm | response to stimulus | 28665.5 | 4.89 | 63 | 4 |
| 694 | Q61696 | Heat shock 70 kDa protein 1A | protein binding | cytoplasm | response to stimulus | 28665.5 | 4.89 | 95 | 4 |
| 962 | Q9JJU8 | SH3 domain-binding glutamic acid-rich-like protein | protein binding | cytoplasm | regulation of biological process | 12803.2 | 4.87 | 80 | 8 |
| 645 | P48036 | Annexin A5 | structural molecule activity | vesicle | response to stimulus | 35599 | 4.82 | 167 | 11 |
| 970 | P10639 | Thioredoxin | antioxidant activity | cytoplasm | regulation of biological process | 11667.6 | 4.8 | 92 | 5 |
| 24 | Q61554 | Fibrillin-1 | structural molecule activity | extracellular region | cell communication | 309208 | 4.79 | 61 | 10 |
| 249 | P09103 | Protein disulfide-isomerase | oxidoreductase activity | cytoplasm | regulation of biological process | 57507 | 4.79 | 84 | 4 |
| 250 | P09103 | Protein disulfide-isomerase | oxidoreductase activity | cytoplasm | regulation of biological process | 57507 | 4.79 | 113 | 9 |
| 252 | P09103 | Protein disulfide-isomerase | oxidoreductase activity | cytoplasm | regulation of biological process | 57507 | 4.79 | 119 | 4 |
| 248 | P09103 | Protein disulfide-isomerase | oxidoreductase activity | cytoplasm | regulation of biological process | 57507 | 4.79 | 145 | 6 |
| 251 | P09103 | Protein disulfide-isomerase | oxidoreductase activity | cytoplasm | regulation of biological process | 57507 | 4.79 | 148 | 4 |
| 434 | Q9D6F9 | Tubulin beta-4A chain | structural molecule activity | cytoskeleton | organelle organization | 49553 | 4.78 | 60 | 6 |
| 866 | Q9CQ19 | Myosin regulatory light polypeptide 9 | structural molecule activity | cytoskeleton | organelle organization | 19854 | 4.78 | 87 | 6 |
| 865 | Q9CQ19 | Myosin regulatory light polypeptide 9 | structural molecule activity | cytoskeleton | organelle organization | 19854 | 4.78 | 92 | 5 |
| 705 | Q9CQV8 | 14-3-3 protein beta/alpha | antioxidant activity | cytoplasm | regulation of biological process | 28068 | 4.77 | 61 | 5 |
| 751 | P35276 | Ras-related protein Rab-3D | nucleotide binding | vesicle | cell communication | 24269 | 4.76 | 61 | 4 |
| 870 | P63028 | Translationally-controlled tumor protein | protein binding | cytoplasm | regulation of biological process | 19449.6 | 4.76 | 80 | 4 |
| 96 | Q91V38 | Heat shock protein 90, beta (Grp94), member 1 | protein binding | cytoplasm | response to stimulus | 92432.3 | 4.74 | 84 | 6 |
| 707 | P63101 | 14-3-3 protein zeta/delta | antioxidant activity | cytoplasm | regulation of biological process | 27925 | 4.73 | 163 | 4 |
| 709 | P63101 | 14-3-3 protein zeta/delta | antioxidant activity | cytoplasm | regulation of biological process | 27753 | 4.73 | 61 | 4 |
| 708 | P63101 | 14-3-3 protein zeta/delta | antioxidant activity | cytoplasm | regulation of biological process | 27753 | 4.73 | 97 | 9 |
| 102 | P08113 | Endoplasmin | protein binding | vesicle | response to stimulus | 90096 | 4.72 | 63 | 7 |
| 99 | P08113 | Endoplasmin | protein binding | vesicle | response to stimulus | 90096 | 4.72 | 102 | 7 |
| 100 | P08113 | Endoplasmin | protein binding | vesicle | response to stimulus | 90096 | 4.72 | 102 | 5 |
| 874 | Q9QVP4 | Myosin regulatory light chain 2 | structural molecule activity | cytoskeleton | organelle organization | 19449 | 4.72 | 69 | 5 |
| 872 | Q9QVP4 | Myosin regulatory light chain 2 | structural molecule activity | cytoskeleton | organelle organization | 19449 | 4.72 | 73 | 5 |
| 604 | Q05816 | Reticulocalbin-1 | ion binding | endoplasmic reticulum | multicellular organismal process | 38089.6 | 4.7 | 65 | 5 |
| 606 | Q05186 | Reticulocalbin-1 | ion binding | endoplasmic reticulum | multicellular organismal process | 38089.6 | 4.7 | 87 | 5 |
| 605 | Q05186 | Reticulocalbin-1 | ion binding | endoplasmic reticulum | multicellular organismal process | 38089.6 | 4.7 | 91 | 7 |
| 662 | P58771 | Tropomyosin alpha-1 chain | structural molecule activity | cytoskeleton | organelle organization | 32681.1 | 4.69 | 85 | 6 |
| 852 | Q99LJ5 | CKLF-like MARVEL transmembrane domain-containing protein 3 | protein binding | membrane | response to stimulus | 20247 | 4.66 | 65 | 4 |
| 701 | Q6IRU2 | Tropomyosin alpha-4 chain | structural molecule activity | cytoskeleton | organelle organization | 28319 | 4.65 | 148 | 4 |
| 685 | P62259 | 14-3-3 protein epsilon | antioxidant activity | cytoplasm | regulation of biological process | 29155 | 4.63 | 68 | 6 |
| 684 | P62259 | 14-3-3 protein epsilon | antioxidant activity | cytoplasm | regulation of biological process | 29155 | 4.63 | 81 | 6 |
| 660 | P58774 | Tropomyosin beta chain | structural molecule activity | cytoskeleton | organelle organization | 32924.6 | 4.61 | 84 | 5 |
| 923 | Q60605 | Myosin light polypeptide 6 | structural molecule activity | cytoskeleton | organelle organization | 16788 | 4.56 | 71 | 5 |
| 924 | Q60605 | Myosin light polypeptide 6 | structural molecule activity | cytoskeleton | organelle organization | 16788 | 4.56 | 88 | 4 |
| 922 | Q642K0 | Myosin light polypeptide 6 | structural molecule activity | cytoskeleton | organelle organization | 16788 | 4.56 | 91 | 6 |
| 877 | Q9D1N2 | lipoprotein Gln I | protein binding | extracellular region | transport | 19171 | 4.54 | 122 | 4 |
| 876 | Q9D1N2 | lipoprotein Gln I | protein binding | extracellular region | transport | 19171 | 4.54 | 124 | 4 |
| 878 | Q9D1N2 | lipoprotein Gln I | protein binding | extracellular region | transport | 19171 | 4.54 | 142 | 4 |
| 942 | O55042 | Alpha-synuclein | structural molecule activity | cytoplasm | regulation of biological process | 14476 | 4.47 | 119 | 4 |
| 444 | P14211 | Calreticulin | protein binding | cytoplasm | regulation of biological process | 47964.9 | 4.33 | 60 | 5 |
| 443 | P14211 | Calreticulin | protein binding | cytoplasm | regulation of biological process | 47964.9 | 4.33 | 91 | 8 |
| 442 | P14211 | Calreticulin | protein binding | cytoplasm | regulation of biological process | 47964.9 | 4.33 | 100 | 5 |
| 121 | P09803 | Cadherin-1 | structural molecule activity | cytoplasm | regulation of biological process | 80267 | 4.28 | 60 | 4 |
| 921 | P62204 | Calmodulin | protein binding | cytoplasm | regulation of biological process | 16883.9 | 4.09 | 87 | 4 |
| 965 | Q80ZC9 | Psoriasis susceptibility 1 candidate gene 1 protein | nucleotide binding | extracellular region | regulation of biological process | 12262 | 3.86 | 63 | 4 |
